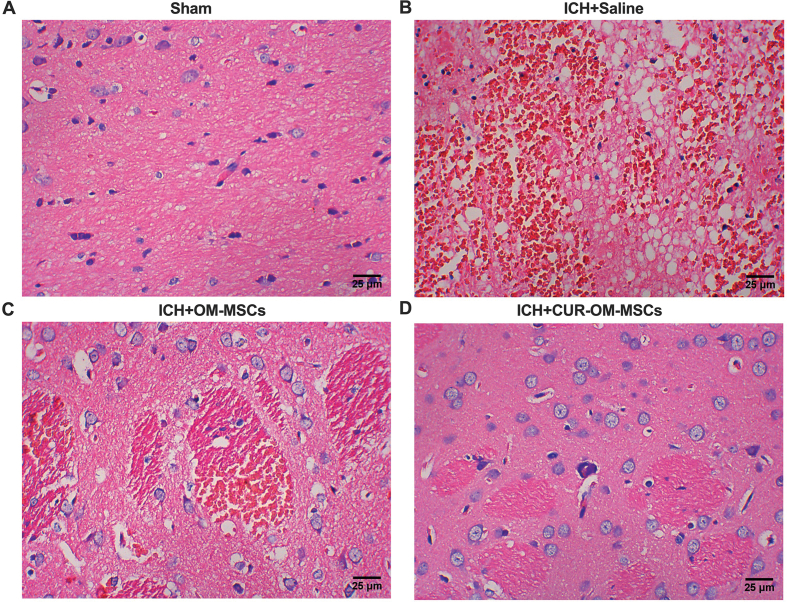# Curcumin preconditioning enhances the neuroprotective effects of olfactory mucosa-derived mesenchymal stem cells on experimental intracerebral hemorrhage

**DOI:** 10.1016/j.heliyon.2023.e17874

**Published:** 2023-07-03

**Authors:** Yan Huang, Jianyang Liu, Jialin He, Fengbo Tan, Ming Lu, Fulai Yuan, Xuelin Zhu, Lingyu Kong

**Affiliations:** a NHC Key Laboratory of Birth Defect for Research and Prevention (Hunan Provincial Maternal and Child Health Care Hospital), Changsha, Hunan 410008, PR China; b Key Laboratory of Protein Chemistry and Developmental Biology of Ministry of Education, College of Life Sciences, Hunan Normal University, Changsha, Hunan 410081, PR China; c Hunan Provincial Key Laboratory of Neurorestoration, PR China; d Department of Neurology, The Second Xiangya Hospital of Central South University, Changsha, Hunan 410011, PR China; e Department of Gastrointestinal Surgery, Xiangya Hospital, Central South University, Changsha, Hunan 410008, PR China; f Health Management Center, Xiangya Hospital, Central South University, Changsha, Hunan 410008, PR China; g Department of Radiology, Xiangya Hospital, Central South University, Changsha, Hunan 410008, PR China; h National Clinical Research Center for Geriatric Disorders, Xiangya Hospital, Central South University, Changsha, Hunan 410008, PR China

**Keywords:** Intracerebral hemorrhage, Mesenchymal stem cells, Curcumin, Preconditioning, Ferroptosis, Oxidative stress

## Abstract

Oxidative stress is essential in brain injury after intracerebral hemorrhage (ICH). Ferroptosis, iron-dependent oxidative cell death, overwhelms the antioxidant system. Recently, Olfactory mucosa-derived mesenchymal stem cells (OM-MSCs) hold great potential for treating ferroptosis-mediated oxidative brain damage after ICH. However, massive grafted cell death, possibly caused by a hostile host brain microenvironment, lessens the effectiveness of OM-MSCs. Therefore, it is necessary to develop strategies to upregulate the therapeutic efficacy of OM-MSCs in ICH. Curcumin, a well-established traditional herbal substance, has potent antioxidant property. In the present study, curcumin preconditioning might enhance the anti-oxidative activity of OM-MSCs, thereby augmenting the therapeutic efficacy of OM-MSCs in ICH. In vitro model of ICH, we demonstrated that curcumin-preconditioned OM-MSCs co-culture is more effective in attenuating the cell injury, oxidative stress, and ferroptosis of neuronal cells compared to the native OM-MSCs treatment. In vivo model of ICH, transplantation of curcumin-preconditioned OM-MSCs also showed better neuroprotective effects. Moreover, curcumin pretreatment promoted the survival of OM-MSCs under a conditioned medium from hemin-insulted neurons by improving the anti-oxidative capacities of OM-MSCs. Collectively, our investigation suggested that curcumin preconditioning effectively enhanced the survival and neuroprotective effects of OM-MSCs in the ICH model by upregulating the anti-oxidative capacities of OM-MSCs. Curcumin-preconditioned OM-MSCs might be taken as a novel therapeutic strategy for treating ICH.

## 1 Introduction

Intracerebral hemorrhage (ICH), also named spontaneous intracerebral hemorrhage, is a severe and devastating cerebral vascular disorder with a high disability and lethality rate [1]. It is well-known that oxidative stress reactive exerts an essential role in the complicated pathological mechanism of brain tissues impairment caused by ICH [2]. Moreover, the exacerbated secondary neurological insult upon ICH condition mainly results from irreversible neuronal death induced by the persistent oxidative stress response. Consequently, the various forms of oxidative cell death might be a potential target for treatment against ICH-triggered neuronal death and neurological function deficit [3]. Recently, a novel subtype of the cell death pathway, named ferroptosis, has been confirmed. Ferroptosis is iron-dependent oxidative cell death featured with intracellular ROS over-excessive generation, which overwhelms the antioxidant protection system, leading to oxidative cell death [4]. Growing research has been demonstrated that ferroptosis occurs in ICH-induced neuronal death [5,6]. Further, applying ferroptosis inhibitor could obviously ameliorate the ICH impairment [7]. Collectively, suppression of ferroptosis in neuronal death might be provided as an available strategy for ICH insult.

Over the past year, given the unsatisfactory therapeutic effect of ICH, exploring and identifying a highly efficacious treatment for ICH is urgently required. Growing reports indicated that the transplantation of Mesenchymal stem cells (MSCs) treatment promises a cure for ICH [8]. Recently, Olfactory mucosa-derived mesenchymal stem cells (OM-MSCs), a novel source of mesenchymal stem cells harvested from olfactory mucosa tissues, have received increasing attention [9]. Compared to the other sources of MSCs, OM-MSCs located in nasal lamina propria originates from the neural crest, rather than traditional sources from mesodermal sources [10]. From identifying the physiological characteristics of OM-MSCs [[11], [12], [13]], we have deeply discussed their potential effect on the therapy of cerebral vascular disorders, including ICH. It was demonstrated that OM-MSCs plays a neuroprotective role either in hemorrhagic or ischemic stroke via regulating oxidative stress, inflammation, and angiogenesis, thereby abolishing various forms of neuronal death, such as apoptosis and pyroptosis and enhancing neurological functional recovery [[14], [15], [16]]. It is worth mentioning that the biological function role of OM-MSCs in ICH-triggered ferroptotic neuronal death has never been reported. Therefore, it is significant to investigate the possible role and molecular mechanism of OM-MSCs on ferroptosis upon ICH-induced brain tissues insult, which might be provided a promising new therapeutic option for treating ICH. However, the complicated pathological microenvironment under ICH condition influences the survival of OM-MSCs, which always restrains the therapeutic efficacy of OM-MSCs transplantation treatment. In addition, compared with the single OM-MSCs transplantation therapy, the combined therapeutic strategies of OM-MSCs might be more reasonable and effective in achieving the curative effect on ICH. Therefore, exploring the novel combination therapy is necessary to further promote the neuroprotective effect of OM-MSCs upon ICH challenge.

Curcumin (CUR) is a well-established traditional herbal substance isolated from the rhizome of Curcuma longa [17]. Several studies have indicated that curcumin significantly affects anti-oxidative, anti-inflammation, and anti-cancer. Duan. et al. reported that curcumin played a critical neuroprotective role *in vitro* and *vivo* models of ICH by inhibiting oxidative stress, cell death, and inflammatory immune response [18]. Whereas, the particular property of curcumin hampered its extensive clinical application in ICH patients due to its poor oral absorption, unsatisfied water solubility, and ineffectively crossing the blood-brain barrier. Hence, accumulated studies have attempted to overcome the disadvantages of curcumin to improve the therapeutic possibilities of curcumin in clinical applications, such as wrapping with nanoparticles, liposomes, and even polymeric [[19], [20], [21]]. However, it remains unknown whether curcumin co-cultured with OM-MSCs could address the deficiencies of curcumin clinical application and improve the cell-based transplantation efficiency of ICH insult.

In the current study, we investigated the functional role and potential mechanism of curcumin combined with OM-MSCs in vivo and *in vitro* models of ICH. We hypothesize that curcumin pretreatment with OM-MSCs could more effectively augment the neuroprotective effects on OM-MSCs upon ICH-initiated brain impairment via suppressing oxidative stress and ferroptosis in neuronal death via promoting the survival and antioxidant capacity of transplanted OM-MSCs under the complicated microenvironment triggered by ICH.

## 2 Material and methods

### 2.1 Isolation and culture of OM-MSCs

Human OM-MSCs were obtained from healthy donors (four males, aged 20–40 years old) at the Second Affiliated Hospital of Hunan Normal University (Changsha, Hunan, China). The ethics committee of the Hunan Normal University has approved this procedure protocol (Approved No.2009163009). The study was conducted in accordance with the Declaration of Helsinki (as revised in 2013). The participants provided their written informed consent to participate in this study. The olfactory mucosal tissue was isolated and cultured following a published protocol [14,22]. OM-MSCs at passages 3 and 4 were used for further experiments.

### 2.2 Curcumin preconditioning

Curcumin (CUR) was purchased from Sigma-Aldrich (St. Louis, MO, USA), prepared in DMSO, and stored at −20 °C. The OM-MSCs were pretreated with CUR at a final concentration of 10 μM in 10% FBS-containing culture medium for 24 h.

### 2.3 Identification of OM-MSCs

The characteristics of the OM-MSCs and CUR-OM-MSCs were identified using PE-conjugated antibodies against specific membrane markers (CD34, CD45, CD73, CD90, and CD105; eBioscience, San Diego, CA, USA) by using a flow cytometer (Beckman, USA).

### 2.4 In vitro experimental design

#### 2.4.1 Primary cortical neurons

The primary cortical neurons were harvested from 16 to 18 days old Sprague-Dawley rat embryos following a previously reported protocol [23].

#### 2.4.2 ICH model *in vitro*

To simulate conditions of the *in vitro* ICH model, 1 × 10^5^ neurons were cultured with Hemin (APExBIO; C3984). To determine the appropriate dose of hemin treatment, neurons were incubated with 0, 25, 50, and 100 μM hemin for 12 h. After filtering the suitable dose, we determine the appropriate hemin treatment duration. Neurons were cultured with Hemin for 0, 6, 12, and 24 h. The viability and lactate dehydrogenase (LDH) release assay of the neuron was assessed using the CCK-8 Assay Kit (Dojindo Molecular Technologies) and LDH Assay Kit (Nanjing Jiancheng Bioengineering Institute, China).

#### 2.4.3 Transwell co-culture of neuron and OM-MSCs

The co-culture was set up by 0.4 μm pore size Transwell plates (Corning Incorporated, China) that allow the diffusion of soluble factors but no cells. For Hemin + OM-MSCs/CUR-OM-MSCs group, 1 × 10^5^ neurons were grown at the bottom of 6-well culture plates and treated with Hemin for 12 h. During the hemin treatment, 1.5 × 10^5^ OM-MSCs/CUR-OM-MSCs were grown on the upper chamber of transwell plate inserts with a pore size of 0.4 μm. The transwell plates were also cultured for 12 h. The relative measurements were performed after hemin treatment.

#### 2.4.4 Preparation of neuron-derived conditioned mediums (CMs)

The C.M.s were grouped into neuron-CMs and hemin-neuron-CMs. Neurons at approximately 80% confluence were washed with non-serum DMEM and then incubated under normal condition, or 50 μM hemin-simulated conditions for 12 h. After 12 h, the C.M.s were collected and immediately centrifuged at 1000 rpm for 10 min.

OM-MSCs/CUR-OM-MSCs at approximately 80% confluence were washed with PBS and then incubated with normal C.M.s, or neuron-CMs, or hemin-neuron-CMs for 12 h. After 12 h, we assessed the viability, LDH release assay, and oxidative stress of OM-MSCs/CUR-OM-MSCs.

#### 2.4.5 Drug treatment

To determine the role of oxidative stress in the survival of OM-MSCs, we incubated OM-MSCs with the oxidative stress-inducing H_2_O_2_ (216,763; Sigma-Aldrich, St Louis, MO, USA), and the antioxidant agent, *N*-acetyl cysteine (NAC, Beyond, China) for 24 h before the treatment with neuron-derived C.M.s.

### 2.5 *In vivo* experimental design

#### 2.5.1 Animals

All animal procedures were performed in accordance with the Guide for the Care and Use of Experimental Animals, and were approved by the Animal Care and Use Committee of Hunan Provincial Maternal and Child Health Care Hospital (Approved No.2021-S010). Male Sprague-Dawley (S.D.) rats (weighing 240–260 g) were kept under controlled housing conditions (12-h light/dark cycle, a constant ambient temperature of 20–25 °C, and humidity of 40–60%).

#### 2.5.2 ICH model in vivo

All rats were randomly divided into four groups: sham-operated group (sham), ICH + saline group, ICH + OM-MSCs group, and ICH + CUR-OM-MSCs group. The rats were anesthetized with 3.5% isoflurane and maintained with 2.0% isoflurane in 2:1 N_2_O/O_2_ using a face mask. Briefly, the ICH model was performed by the injection of collagenase IV (Sigma-Aldrich) (0.5 U dissolved in 2.0 μL saline) at the rate of 0.1 μL/min. The position: 0.2 mm anterior, 3.0 mm lateral, and 6.0 mm ventral bregma. The sham group underwent the same procedures without the collagenase type IV injection.

#### 2.5.3 OM-MSCs intracerebral transplantation

The surviving animals were randomized into the OM-MSCs or CUR-OM-MSCs group at 6 h post-ICH surgery. For the ICH + OM-MSCs/CUR-OM-MSCs treated group, 1.5 × 10^6^ OM-MSCs/CUR-OM-MSCs in 10 μL saline were stereotactically transplanted into the edge of ipsilateral lesion area. For the ICH + saline-treated group, 10 μL saline was administrated to the same position. Experimental rats were sacrificed at 24 h post-ICH model.

### 2.6 Cell apoptosis assay

Apoptosis of neurons was measured via the FITC Annexin V apoptosis detection kit (KGA108, KeyGen Biotech, China) according to the manufacturer's instructions.

### 2.7 Measurement of intracellular ROS generation

The change of intracellular ROS in neurons and OM-MSCs was measured using the semiquantitative dichlorfluorescein-diacetate (DCFH-DA, Beyotime Biotechnology) according to manufacturer's instruction and then documented by flow cytometer (Becton Dickinson, CA, USA).

### 2.8 Measurement of ATP, MDA, LPO, T-AOC, T-GSH, and GSH/GSSG activity

The supernatant of neuron and OM-MSCs, and the peri-hematoma cortex homogenates (10% wt/vol) was used for the analysis of malondialdehyde (MDA), lipid peroxidase (LPO), total antioxidant capacity (T-AOC), total glutathione (T-GSH), glutathione/Oxidized glutathione (GSH/GSSG) levels. The level was determined by the commercial kits (Nanjing Jiancheng Biotech, China).

### 2.9 Measurement of GPX4 activity

The levels of GPX4 activities in the neuron and the peri-hematoma cortex homogenates were determined using the GPX4 activity assay kit (CUSABIO, Wuhan, China).

### 2.10 Measurement of iron content

The iron assay kit determined the levels of Fe^2+^ and total iron in the neuron and the peri-hematoma cortex homogenates (Abcam, Cambridge, MA).

### 2.11 Western blot analysis

Proteins were extracted from the neuron and the peri-hematoma cortex tissue as described previously [24]. Immunoblot analyses were performed using the following primary antibodies: cleaved-caspase3 (1:500; ab49822; abcam), GP4X (1:750; 14432-1-AP; proteintech), FTH1 (1 μg/ml; ab65080; abcam), ACSL-4 (1:100; 22401-1-AP; proteintech), SLC7A11 (1:1000; 26864-1-AP; proteintech), occludin (1:1000; 27260-1-AP; proteintech), claudin-5 (1:5000; ab131259; abcam), ZO-1 (1:5000; 21773-1-AP; proteintech), β-actin (1:5000; 66009-1-Ig; proteintech) at 4 °C overnight. Secondary antibodies included anti-rabbit IgG and anti-mouse IgG (1: 5000; SA00001-1; SA00001-2; proteintech). The proteins were visualized using the ChemiDoc XRS imaging system.

### 2.12 Neurobehavioral tests

The Garcia score [25] was used to quantify neurological function before and at 24 h, 48 h, and 72 h after ICH with different treatments. The behavioral test was blindly performed to assess neurological deficiency.

### 2.13 Brain water content

Rats were decapitated under deep anesthesia at 24 h after ICH, and the brains were immediately removed. The ipsilateral hemispheres were immediately weighed using an electric analytic balance to obtain the wet weight. The samples were then dried at 100 °C for 24 h, and the dry weight was obtained. Brain water content was calculated as brain water content (%) = (wet weight - dry weight)/wet weight × 100%.

### 2.14 Evan's blue dye extravasation assay

The Evan's Blue dye extravasation assay was measured at 24 h post-ICH. Rats were intravenously administered with Evans blue solution (Sigma-Aldrich) at 3 h before being sacrificed. The ipsilateral hemispheres were homogenized in N, *N*-dimethylformamide and centrifuged. The supernatant was measured by spectrophotometry at the wavelength of 620 nm.

### 2.15 Immunofluorescence and TUNEL staining

At 24 h after the ICH model, the brain of rats was extracted, and coronal sections were obtained at a thickness of 10 μm (Leica CM1850). The rate of apoptotic cells in the peri-hematoma brain tissues were assessed by the terminal deoxynucleotidyl transferase biotin-mediated dUTP Nick-end labeling (TUNEL) staining (Promega Corporation, United States). Nuclei were stained with 6-diamidino-2-phenylindole (DAPI; Sigma). The slides were observed under a fluorescent microscope.

The ferroptotic neuronal death in peri-hematoma brain tissues were examined by the double staining of GPX4 and neuronal marker NEUN. Brain sections were incubated with primary antibodies, including *anti*-GPX4 (1:50; 14432-1-AP; Proteintech), and *anti*-NEUN (1:50; ab104224; Abcam) overnight at 4 °C. After being washed with PBS three times, the slices were incubated with fluorescence-conjugated secondary antibodies (1:500, Proteintech) for 2 h, followed by staining with DAPI.

### 2.16 Hematoxylin-eosin (HE) staining

At 24 h after ICH induction, animals were decapitated under deep anesthesia and underwent *trans*-cardiac perfusion with 4% paraformaldehyde. The sections of brain sections were deparaffinized with graded ethanol together with xylene, and 4 μm sections were prepared for hematoxylin-eosin staining. After being dehydrated with graded ethanol and xylene, the histological structure was observed under a light microscope (Motic, BA210T, China).

### 2.17 Transmission electron microscope (TEM)

The cortex of the perihematomal region from the fresh brain samples were separated and embedded in epoxy resin. The observations were carried out using an electron microscope (Hitachi, HT7700, Japan).

### 2.18 Statistical analysis

The results are expressed as mean ± standard error of mean (SEM). Comparisons among groups were estimated using two-sided unpaired Student's t-test or ANOVA with the Bonferroni correction for the post hoc *t*-test as appropriate. Two-way ANOVA was adopted for the data of different treatment groups and multiple time points. Statistical analysis was calculated using GraphPad Prism 6 Software. Values of P < 0.05 were considered significant.

## 3 Results

### 3.1 Identification of OM-MSCs and CUR-OM-MSCs

To obtain curcumin preconditioned OM-MSCs, different concentrations of curcumin were co-incubated with OM-MSCs first. We found that the concentration of curcumin higher than 10 μM significantly impaired the activity of OM-MSCs (Supplementary Figure 1; *p* < 0.001). Therefore, the optimum concentration of curcumin treating OM-MSCs was 10 μM, which was used in the following research.

Flow cytometry identified surface markers expressions, which were positive for CD73, CD90, and CD105, but negative for CD34 and CD45. There was no significant difference in surface markers between the OM-MSCs and curcumin preconditioned OM-MSCs (CUR-OM-MSCs) (Fig. 1A and B, and Supplementary Figure 2).

**Fig. 1:**
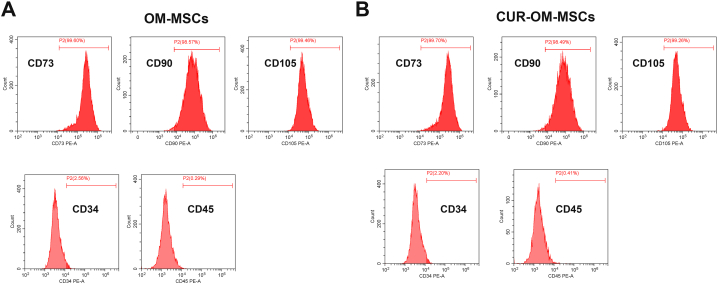
**Curcumin (CUR)-preconditioned olfactory mucosa-derived mesenchymal stem cells (OM-MSCs) maintain the stem cell properties.** (**A**) The flow cytometry assesses for the immunophenotypic marker of OM-MSCs. (**B**) The flow cytometry assesses for the immunophenotypic marker of CUR-OM-MSCs.

### 3.2 CUR-OM-MSCs co-culture attenuated the cell injury of neuronal cells in response to hemin *in vitro*

To determine whether OM-MSCs exerted protective effects on neuronal cells in an *in vitro* ICH model, we detected the viability, toxicity, and apoptosis of hemin-treated neuronal cells under the transwell co-culture with OM-MSCs/CUR-OM-MSCs. First, we examined the neuronal cells viability under different doses of hemin treatment. The results showed that neuronal viability decreased in Hemin dose-dependent manner, and subsequent experiments were carried out with 50 μM Hemin as this concentration significantly increased cell mortality (Supplementary Figure 3; *p* < 0.001). Second, we examined the neuronal cells injury under the different duration of hemin treatment. After treatment with 50 μM Hemin, cell viability and LDH level changed in a time-dependent manner, reaching a minimum and maximum at 12 h (Fig. 2A–B; *p* < 0.001, *p* < 0.01, respectively). Thus, we chose the 12 h of treatment time for the following research.

**Fig. 2:**
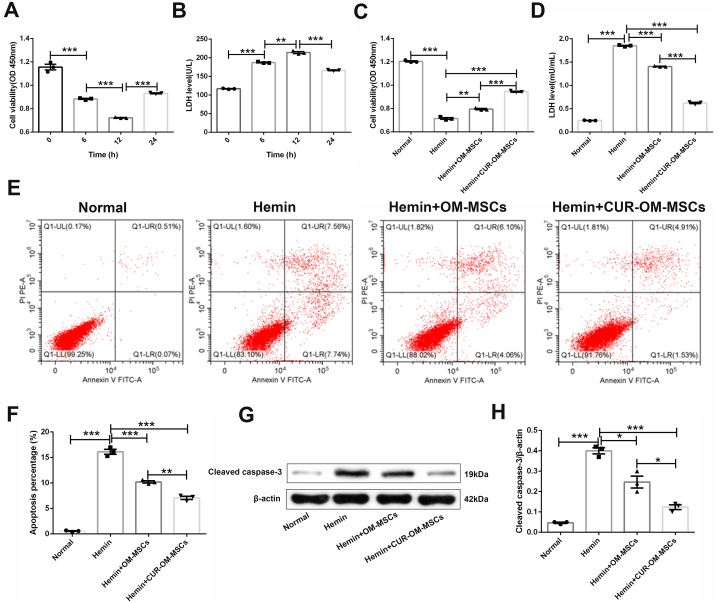
**CUR-OM-MSCs co-culture attenuates the cell injury of neurons in response to Hemin *in vitro*.** (**A**) The cell viability of neurons treated with Hemin for different lengths of time. (**B**) The LDH level of neurons treated with Hemin for different lengths of time. (**C**) The cell viability of neurons treated with Hemin and co-cultured with OM-MSCs/CUR-OM-MSCs. (**D**) The LDH level of neurons treated with Hemin and co-cultured with OM-MSCs/CUR-OM-MSCs. (**E-F**) The cell apoptosis of neurons treated with Hemin and co-cultured with OM-MSCs/CUR-OM-MSCs. (**G-H**) Western blotting for cleaved caspase-3 in neurons treated with Hemin and co-cultured with OM-MSCs/CUR-OM-MSCs. Data are expressed as the mean ± SEM (n = 3), *P < 0.05, **P < 0.01, ***P < 0.001. The raw data of Fig. 2G is presented inSupplementary Fig. 6.

In the *in vitro* model, co-culture with both OM-MSCs and CUR-OM-MSCs significantly improved the morphology (Supplementary Figure 4), enhanced the cell viability (Fig. 2C; 0.795 ± 0.006 vs 0.715 ± 0.007 *p* < 0.01, 0.945 ± 0.004 vs 0.715 ± 0.007 *p* < 0.001, respectively) and reduced the LDH level (Fig. 2D; 1.407 ± 0.007 vs 1.850 ± 0.010, 0.624 ± 0.016 vs 1.850 ± 0.010, *p* < 0.001) of the insulted neuronal cells. Moreover, its protective effect on the injury neuronal cell was more remarked in the CUR-OM-MSCs group than in the OM-MSCs alone group. In the detection of cell apoptosis, flow cytometry showed a lower percentage of apoptotic cells in the CUR-OM-MSCs co-cultured group compared with the native OM-MSCs co-cultured group (Fig. 2E–F; 7.077% ± 0.325% vs 10.213% ± 0.221%, *p* < 0.01). Meanwhile, apoptosis-related protein cleaved caspase-3 within neuronal cells showed obviously downregulated expression in CUR-OM-MSCs co-cultured group, compared with the native OM-MSCs co-cultured group (Fig. 2G–H; 0.123 ± 0.012 vs 0.247 ± 0.029, *p* < 0.05). Collectively, these results indicated that CUR-OM-MSCs co-culture is more effective in attenuating the cell injury of neuronal cells under the *in vitro* ICH model.

### 3.3 CUR-OM-MSCs pretreatment exhibited enhanced anti-oxidative effects of the insulted neuronal cells induced by hemin *in vitro*

Given the essential role of oxidative stress on the mechanism after ICH, the level of oxidative stress was assessed with intracellular ROS, ATP, LPO, MDA, T-AOC, T-SOD, T-GSH, and GSH/GSSG indexes in neuronal cells. The flow cytometry showed that the level of intracellular ROS in hemin-treated neuronal cells was significantly down-regulated in the OM-MSCs and CUR-OM-MSCs co-cultured group (Fig. 3A; 5868.733 ± 51.505 vs 10125.600 ± 84.617, 3720.000 ± 26.412 vs 10125.600 ± 84.617, *p* < 0.001). Further, there was a marked reduction in the CUR-OM-MSCs group compared with OM-MSCs alone group in the damaged neuronal cells caused by Hemin (Fig. 3A; *p* < 0.001). Except for the ROS production, the ATP, T-AOC, and oxidant-related enzymes were evaluated in hemin-treated neuronal cells. Compared with the hemin group, the ATP, T-AOC, T-SOD, T-GSH, and GSH/GSSG levels of the impaired neuronal cells were significantly raised in the OM-MSCs and CUR-OM-MSCs co-cultured group (Fig. 3B, 3 E-3H; *p* < 0.05). Additionally, there was an obviously decreased level of the oxidative stress under hemin conditions in the CUR-OM-MSCs treated group relative to the OM-MSCs treated group (Fig. 3B, 3 E-3H; *p* < 0.05). Similarly, the detection of LPO and MDA levels also indicated that OM-MSCs co-culture, especially the CUR-OM-MSCs, reduced the oxidative stress in neuronal cells (Fig. 3C–D; *p* < 0.05). Collectively, these results demonstrated that CUR-preconditioning OM-MSCs more obviously enhanced the anti-oxidative effects of the insulted neuronal cells exposed to Hemin.

**Fig. 3:**
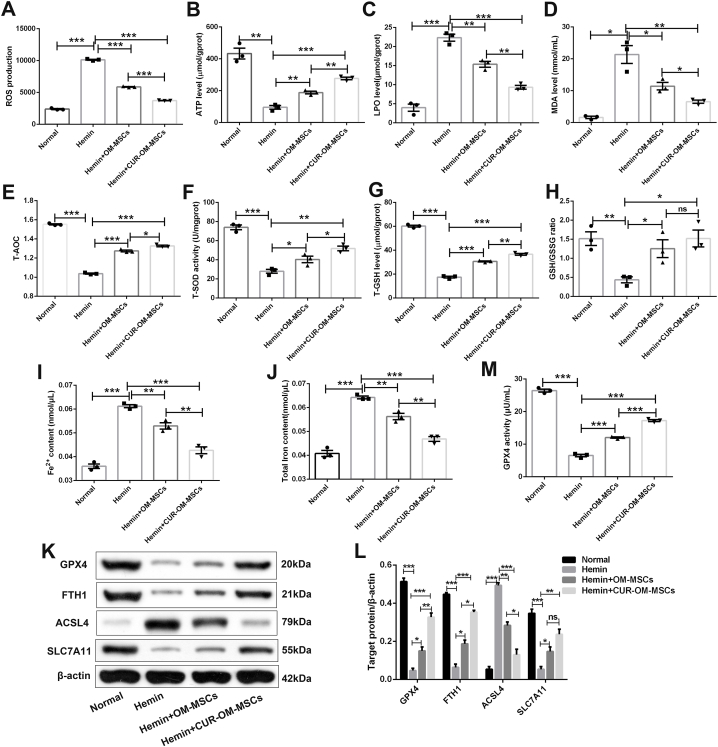
**CUR-OM-MSCs inhibit oxidative stress and attenuate ferroptosis of Neurons *in vitro.*** (**A-H**) The levels of reactive oxygen species (ROS), adenosine triphosphate (ATP), lipid hydroperoxide (LPO), malonaldehyde (MDA), total antioxidant capacity (T-AOC), total superoxide dismutase (T-SOD), total glutathione (T-GSH), and GSH/oxidized glutathione (GSSG) ratio in neurons treated with Hemin and co-cultured with OM-MSCs/CUR-OM-MSCs. (**I-J**) The contents of Fe^2+^ and total iron in neurons treated with Hemin and co-cultured with OM-MSCs/CUR-OM-MSCs. (**K-L**) Western blotting for GPX4, FTH1, ACSL4, and SLC7A11 in neurons treated with Hemin and co-cultured with OM-MSCs/CUR-OM-MSCs. (**M**) The GPX4 activity of neurons treated with Hemin and co-cultured with OM-MSCs/CUR-OM-MSCs. Data are expressed as the mean ± SEM (n = 3), *P < 0.05, **P < 0.01, ***P < 0.001, ns, no significance. The raw data of Fig. 3K is presented in Supplementary Fig. 6.

### 3.4 CUR-OM-MSCs attenuated hemin-triggered ferroptosis in neuronal cells *in vitro*

Considering ferroptosis is a form of oxidative programmed cell death, we continue to explore the effect of CUR-OM-MSCs on Hemin-triggered ferroptosis in neuronal cells.

First, we evaluated the changes in iron levels in neuronal cells after hemin insult. Compared to the normal group, Fe^2+^ and total iron levels were increased in the hemin group. Both OM-MSCs and CUR-OM-MSCs co-culture markedly reduced the Fe^2+^ and total iron levels, and the decrease was more significant with the CUR-OM-MSCs group treatment group (Fig. 3I–J; 0.043 ± 0.001 vs 0.053 ± 0.001, 0.047 ± 0.001 vs 0.056 ± 0.001, *p* < 0.01). Then, the protein expressions of negative mediators (GPX4, FTH1, and SLC7A11) and positive mediator (ACSL4) of ferroptosis were assayed. The Western blot showed that co-culture with OM-MSCs and CUR-OM-MSCs significantly upregulated the negative mediators, and down-regulated the positive mediator compared to the hemin-treated neuron group. Compared to the OM-MSCs alone group, the improvement of GPX4, FTH1, and ACSL4 in CUR-OM-MSCs was remarkedly higher (Fig. 3K–L). In addition, the GPX4 activity by ELISA was further confirmed, which also showed that CUR-OM-MSCs produce significantly more promotion than OM-MSCs alone treatment (Fig. 3M; 17.251 ± 0.282 vs 12.040 ± 0.190, *p* < 0.001).

Taken together, the above results indicated that CUR-OM-MSCs could more remarkably rescue the damaged neuronal cells caused by Hemin by enhancing anti-oxidative stress capacity and cell viability, mitigating ferroptosis and apoptosis compared to a single OM-MSCs therapy *in vitro* of ICH model. Hence, the subsequent assays identified whether CUR-OM-MSCs have a more neuroprotective role in the ICH model in vivo.

### 3.5 Administration of CUR-OM-MSCs mitigated the levels of oxidative stress in the damaged peri-hematoma brain tissues caused by the ICH model in vivo

OM-MSCs/CUR-OM-MSCs were stereotactically transplanted into the area surrounding the hematoma 6 h post-ICH. To assess the levels of oxidative stress of OM-MSCs and CUR-OM-MSCs, we also detected the total antioxidant capacity and oxidant-associated enzymes in peri-hematoma brain tissue at 24 h post-ICH. The flow cytometry showed that the upregulated level of ROS in ICH-treated peri-hematoma brain tissue was significantly reduced in the OM-MSCs and CUR-OM-MSCs co-cultured group. Moreover, there was a marked reduction in the CUR-OM-MSCs group compared with OM-MSCs alone group in the peri-hematoma brain tissue after ICH insult (Fig. 4A; 5574.080 ± 337.421 vs 8353.480 ± 238.349, *p* < 0.001). Compared with the ICH + Saline group, the ATP, T-AOC, T-SOD, T-GSH, and GSH/GSSG levels were significantly promoted in the OM-MSCs and CUR-OM-MSCs transplantation group in the peri-hematoma brain tissue after ICH insult (Fig. 4B, 4 E-4H; *p* < 0.05). Further, there was a more remarkable improvement in the CUR-OM-MSCs therapy group relative to the OM-MSCs treatment group upon ICH surgery. Similarly, compared to the ICH + Saline group, the apparent reduction of LPO and MDA levels were observed in the OM-MSCs and CUR-OM-MSCs transplantation group. As expected, the CUR-OM-MSCs group exhibited significantly lower LPO and MDA levels in peri-hematoma brain tissue than the OM-MSCs group upon ICH condition (Fig. 4C–D; *p* < 0.01). Collectively, these results demonstrated that CUR-OM-MSCs administration presented a significant role in suppressing the levels of oxidative stress in the damaged peri-hematoma brain tissues of the ICH model via modulating anti-oxidative capacity and oxidant-associated enzymes. Next, we evaluated the functional role of CUR-OM-MSCs in ferroptotic neuronal death in the ICH model in vivo.

**Fig. 4:**
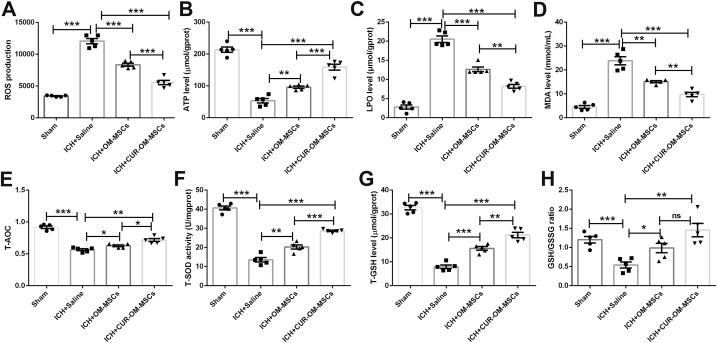
**Administration of CUR-OM-MSCs mitigated the levels of oxidative stress in the damaged peri-hematoma brain tissues caused by the intracerebral hemorrhage (ICH) model *in vivo*.** (**A-H**) The levels of ROS, ATP, LPO, MDA, T-AOC, T-SOD, T-GSH, GSH/GSSG of peri-hematoma tissue after ICH. Data are expressed as the mean ± SEM (n = 5/group), *P < 0.05, **P < 0.01, ***P < 0.001, ns, no significance.

### 3.6 Administration of CUR-OM-MSCs reduced ferroptotic neuronal death in peri-hematoma brain tissues triggered by ICH in vivo

**Fig. 5:**
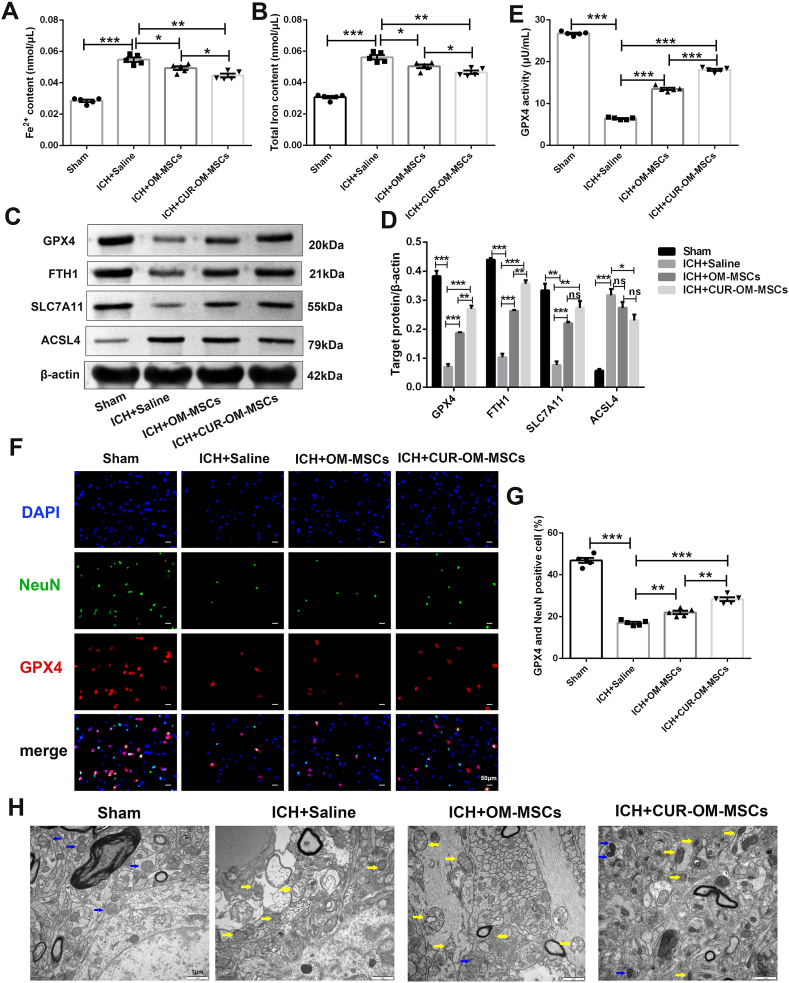
**Administration of CUR-OM-MSCs reduces ferroptotic neuronal death *in vivo*.** (**A-B**) The contents of Fe^2+^ and total iron in peri-hematoma cortex tissue after ICH. Data are expressed as the mean ± SEM (n = 5/group), *P < 0.05, **P < 0.01, ***P < 0.001. (***C*-D**) Western blotting for GPX4, FTH1, ACSL4, and SLC7A11 in peri-hematoma cortex tissue after ICH. Data are expressed as the mean ± SEM (n = 3/group), *P < 0.05, **P < 0.01, ***P < 0.001, ns, no significance. (**E**) The GPX4 activity of peri-hematoma cortex tissue following ICH. (**F**) Representative images of GPX4 and NeuN double staining in ipsilateral cortex after ICH. (**G**) Quantification of GPX4 and NeuN double stained cells. Data are expressed as the mean ± SEM (n = 5/group), **P < 0.01, ***P < 0.001. (**H**) The ultrastructural features of the cortex of perihematomal region as demonstrated by transmission electron microscope. The raw data of Fig. 5C is presented inSupplementary Fig. 6.

To further investigate whether CUR-OM-MSCs transplantation could ameliorate ferroptotic neuronal death at 24 h following ICH, the Fe^2+^ content and total iron levels were further defined in the peri-hematoma cortex tissue caused by ICH surgery. Compared with the sham-operated group, both Fe^2+^ and total iron levels were remarkably raised in the ICH + Saline group but were significantly restored toward normal levels after OM-MSCs administration. Further suppression was observed in those administrated with CUR-OM-MSCs (Fig. 5A–B; 0.045 ± 0.001 vs 0.049 ± 0.001, 0.046 ± 0.001 vs 0.050 ± 0.001, *p* < 0.05). The protein expressions of negative mediators (GPX4, FTH1, and SLC7A11) were significantly elevated in the OM-MSCs and CUR-OM-MSCs transplantation group, compared with the ICH + Saline group. Compared to the OM-MSCs group, the protein expressions of GPX4 and FTH1 were further upregulated in the CUR-OM-MSCs group. On the contrary, the level of the positive mediator of ACSL4 expression was remarkably suppressed in the CUR-OM-MSCs transplantation group, relative to the ICH + Saline group (Fig. 5C–D; 0.230 ± 0.021 vs 0.317 ± 0.022, *p* < 0.05). Further, GPX4 activities detected by ELISA were also decreased in the ICH + Saline group, while these trends were partially reversed after OM-MSCs and CUR-OM-MSCs treatment. The GPX4 activity was obviously enhanced in the CUR-OM-MSCs group than in the native OM-MSCs group (Fig. 5E; 18.052 ± 0.249 vs 13.522 ± 0.277, *p* < 0.001). Meanwhile, the above experimental results were further verified by immunofluorescence detection via double staining of GPX4 and neuronal marker NEUN. The images exhibited that the co-localization of NEUN and GPX4 significantly deregulated in the ICH + saline group, while both in the OM-MSCs and CUR-OM-MSCs transplantation group totally obviously improved the distribution of GPX4 in neurons, and the improvement was more pronounced in CUR-OM-MSCs group relative to the OM-MSCs group (Fig. 5F–G; 28.388% ± 0.917% vs 22.020% ± 0.835%, *p* < 0.01).

To establish whether OM-MSCs transplantation improves ultrastructural features of neuronal ferroptosis, we performed transmission electron microscopy of the cortex of the perihematomal region. In cortical neurons following ICH, the dense and smaller mitochondria were more prevalent than in control neurons, and this trend was dramatically abrogated by OM-MSCs transplantation (Fig. 5H).

Taken together, these results demonstrated that CUR-OM-MSCs treatment exhibited much stronger ameliorative ferroptotic neuronal death caused by ICH injury in peri-hematoma brain tissues. Therefore, the subsequent experiments determined whether CUR-OM-MSCs transplantation could exert a more neuroprotective effect on recovering the impairment of peri-hematoma brain tissues upon ICH condition in vivo.

### 3.7 Administration of CUR-OM-MSCs alleviated the blood-brain barrier dysfunction in peri-hematoma brain tissues induced by ICH in *vivo*

**Fig. 6:**
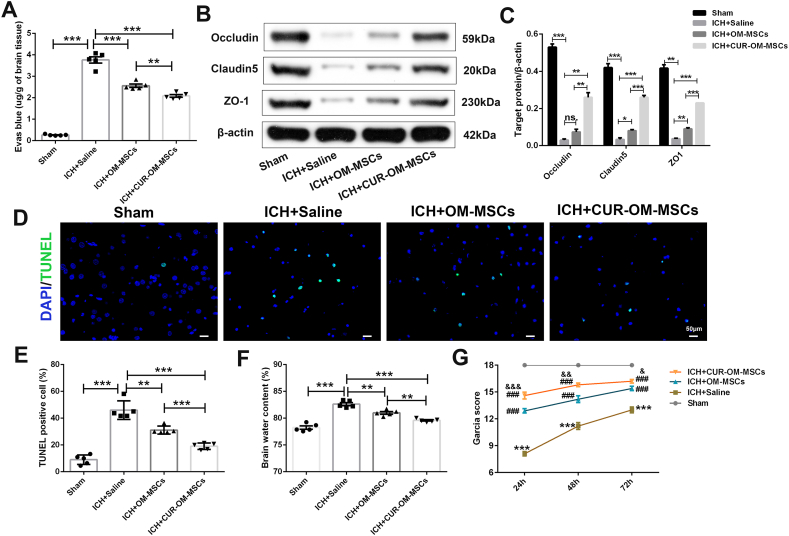
**Administration of CUR-OM-MSCs alleviates blood brain barrier (BBB) dysfunction and shows better neuroprotective effects *in vivo*.** (**A**) The Evan's Blue dye extravasation assay of ipsilateral hemispheres after ICH. Data are expressed as the mean ± SEM (n = 5/group), **P < 0.01, ***P < 0.001. (**B–C**) Western blotting for Occludin, Claudin5, and ZO-1 in the peri-hematoma cortex tissue after ICH. Data are expressed as the mean ± SEM (n = 3/group), *P < 0.05, **P < 0.01, ***P < 0.001, ns, no significance. (**D**) Representative images of terminal transferase-mediated dUTP nick end labeling (TUNEL) staining in ipsilateral cortex after ICH. (**E**) Quantification of TUNEL positive cells. (**F**) Brain water content of ipsilateral hemispheres after ICH. Data are expressed as the mean ± SEM (n = 5/group), **P < 0.01, ***P < 0.001. (**G**) The Garcia score test is performed at 24, 48, and 72 h after ICH. Data are expressed as the mean ± SEM (n = 10/group at 24 h; n = 5/group at 48 and 72 h), ***P < 0.001, vs. sham group; ###P < 0.01 vs. ICH + saline group; &P < 0.05, && P < 0.01, &&&P < 0.001 vs. ICH + OM-MSCs group. The raw data of Fig. 6B is presented inSupplementary Fig. 6.

Considering that BBB dysfunction is one of the essential causes of brain edema post-ICH in brain insult, we assessed the BBB permeability 24 h post-ICH. The intensity of Evan's blue determined by spectrofluorometric estimation showed that BBB permeability in the ICH + Saline group was significantly enhanced compared with the sham-operated group. OM-MSCs transplantation reduced BBB permeability. After being pretreated with curcumin, CUR-OM-MSCs transplantation showed improved BBB permeability post-ICH improvements relative to the OM-MSCs therapy group (Fig. 6A; 2.088 ± 0.062 vs 2.560 ± 0.078, *p* < 0.01).

The Western blot results also showed that the expression levels of BBB integrity markers (Occludin, Claudin5, and ZO-1) were significantly decreased in the ICH + saline group. However, the expressions of Claudin5 and ZO-1 in the OM-MSCs/CUR-OM-MSCs transplantation group were significantly increased, especially in the CUR-OM-MSCs group. For the presentations of Occludin, only CUR-OM-MSCs treatment partially reversed the down-regulation after ICH (Fig. 6B–C). Collectively, these results suggested that CUR-OM-MSCs transplantation remarkedly enhanced the recovery of BBB integrity after ICH impairment in peri-hematoma brain tissues**.**

### 3.8 Administration of CUR-OM-MSCs showed better neuroprotective effects in the in vivo ICH model

To further investigate the neuroprotective effects of MSCs and CUR-OM-MSCs in the ICH-damaged brain tissues, a series of tests, including TUNEL assay, HE staining, brain water content, and Garcia scores, were conducted post-ICH. TUNEL staining was used to examine cell apoptosis in the perihematomal region of rats at 24 h post-ICH (Fig. 6D). Compared with the sham-operated group, the ICH + Saline group exhibited a significantly elevated apoptosis rate in peri-hematoma brain tissues induced by ICH (46.054% ± 3.098% vs 9.054% ± 1.584%, *p* < 0.01). Whereas the apoptosis rate was obviously inhibited in the OM-MSCs and CUR-OM-MSCs transplantation group and mediated a more reduction in the CUR-OM-MSCs treatment group (Fig. 6E; 19.068% ± 1.054% vs 31.124% ± 1.320%, *p* < 0.01). The HE staining of brain tissues surrounding the hematoma suggested remarkable histological impairments in the ICH + saline group, intervention with OM-MSCs, especially the CUR-OM-MSCs notably ameliorated the histological damages in ICH rats (Supplementary Figure 5). Moreover, the brain water content at 24 h post-ICH was measured post-ICH insult. The data found that compared with the sham-operated group, brain edema was remarkedly raised in the ICH + Saline group (82.63% ± 0.285% vs 78.252% ± 0.314%, *p* < 0.001), while the OM-MSCs and CUR-OM-MSCs transplantation group were obviously ameliorated the symptoms of brain edema. Compared with the OM-MSCs alone group, the brain water content was further decreased in the CUR-OM-MSCs transplantation group (Fig. 6F; 79.588% ± 0.135% vs 80.988% ± 0.244%, *p* < 0.01). Before measuring the TUNEL assay, HE staining, and brain water content, the neurobehavioral function by Garcia scores was evaluated. Compared with the ICH + Saline group, the OM-MSC groups, particularly the CUR-OM-MSCs group, showed significant improvements in the Garcia scores at 24, 48, and 72 h after ICH (Fig. 6G).

Taken together, the above data indicated that curcumin combined with OM-MSCs transplantation therapy could effectively prevent against the excessive oxidative stress as well as ferroptosis neuronal death caused by ICH injury, thereby rescuing brain tissues impairment and neurological deficit post-ICH insult. Moreover, this novel combination strategy is a potential candidate therapy for future clinical application in the ICH challenge.

### 3.9 Curcumin pretreatment improved the survival and ameliorated the oxidative stress of OM-MSCs subjected to conditioned mediums from hemin-insulted neurons

**Fig. 7:**
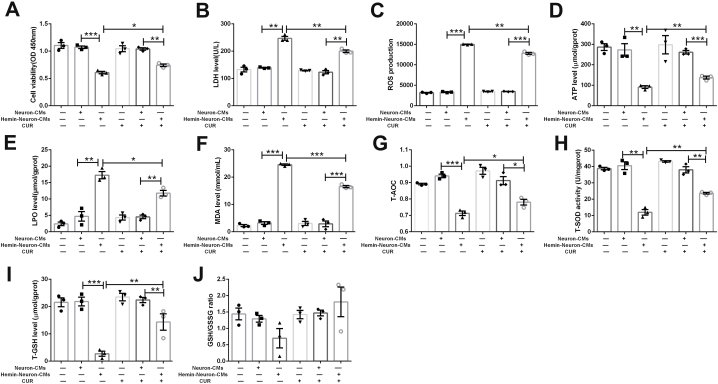
**Curcumin pretreatment improved the survival and ameliorated the oxidative stress of OM-MSCs subjected to conditioned mediums (C.M.s) from hemin-insulted neurons.** (**A**) The cell viability of OM-MSCs and CUR-OM-MSCs treated with Neuron-CMs and Hemin-Neuron-CMs. (**B**) The LDH level of OM-MSCs and CUR-OM-MSCs treated with Neuron-CMs and Hemin-Neuron-CMs. (***C*-J**) The levels of oxidative stress in OM-MSCs and CUR-OM-MSCs treated with Neuron-CMs and Hemin-Neuron-CMs, (**C**) ROS, (**D**) ATP, (**E**) LPO, (**F**) MDA, (**G**) T-AOC, (**H**) T-SOD, (**I**) T-GSH, (**J**) GSH/GSSG ratio. Data are expressed as the mean ± SEM (n = 3), *P < 0.05, **P < 0.01, ***P < 0.001.

Given the significantly improved therapeutic efficiency of CUR-OM-MSCs compared with OM-MSCs in the model of ICH, we hypothesized that the significant neuroprotective effects of CUR-OM-MSCs may be related to the increased survival of OM-MSCs with curcumin pretreatment. To further explore the impact of curcumin pretreatment on the survival of OM-MSCs under the complex microenvironment triggered by ICH, we compared the viability and toxicity of OM-MSCs and CUR-OM-MSCs exposed to C.M.s from the hemin-insulted neuron. In contrast with normal neuron-derived C.M.s, treatment with C.M.s from hemin-insulted neurons significantly reduced the cell viability (Fig. 7A; 0.607 ± 0.023 vs 1.070 ± 0.024, *p* < 0.001) and upregulated the LDH level (Fig. 7B; 246.953 ± 7.079 vs 139.128 ± 1.378, *p* < 0.01) of OM-MSCs. However, curcumin pretreatment partially improved the viability and inhibited the LDH level of OM-MSCs. Therefore, curcumin pretreatment promoted the survival of OM-MSCs under the condition of C.M.s from injured neurons.

Considering that curcumin has exerted a significant effect on anti-oxidative in previous studies, we suggested that curcumin may increase the survival of OM-MSCs through inhibition of oxidative stress. Thus, we further detected the oxidative stress-associated indexes of OM-MSCs and CUR-OM-MSCs. The flow cytometry showed that the level of intracellular ROS in OM-MSCs was significantly elevated with the treatment of hemin-neuron-CMs. But there was a marked reduction in the CUR-OM-MSCs group compared with the OM-MSCs group under the microenvironment caused by hemin-insulted neurons (Fig. 7C; 12790.767 ± 233.610 vs 15002.033 ± 56.983, *p* < 0.01). Besides ROS production, ATP and oxidant/antioxidant-related enzymes were also assessed. Compared with neuron-CMs, the ATP, T-AOC, T-SOD, and T-GSH levels of OM-MSCs in the hemin-neuron-CMs group were significantly decreased (Fig. 7D, 7G-7I; *p* < 0.01). No significance was found in the level of GSH/GSSG (Fig. 7J; *p* > 0.05). Similarly, the detection of LPO and MDA levels also indicated that hemin-neuron-CM treatment upregulated the oxidative stress in OM-MSCs (Fig. 7E–F; *p* < 0.01). Additionally, the above oxidant/antioxidant-related enzymes presented an obvious reverse in the CUR-OM-MSCs group relative to the OM-MSCs group (Fig. 7D–I; *p* < 0.05). These findings showed that the curcumin pretreatment elevated the anti-oxidative capacities of OM-MSCs under the condition of C.M.s from hemin-insulted neurons.

### 3.10 Curcumin pretreatment promoted the survival of OM-MSCs by elevating the anti-oxidative capacities

**Fig. 8:**
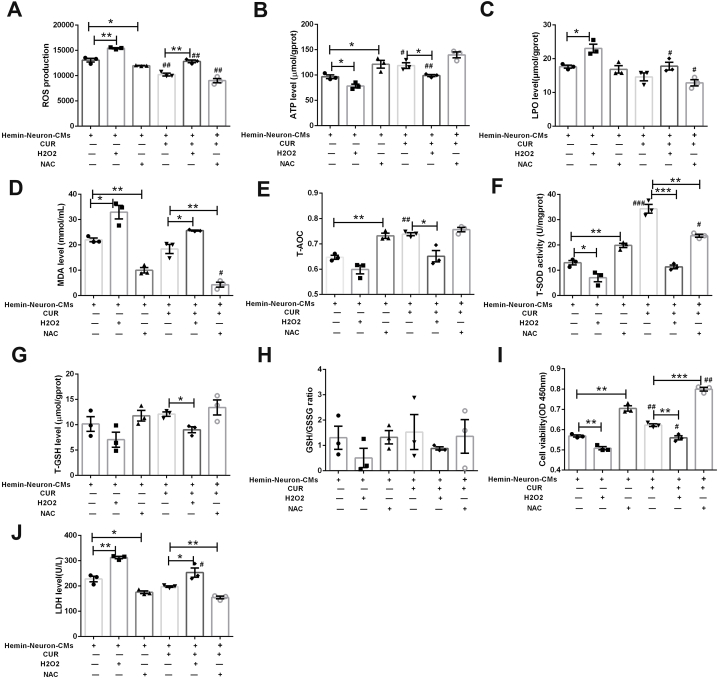
**Curcumin pretreatment promotes the survival of OM-MSCs via elevating the anti-oxidative capacities.** H_2_O_2_ and NAC are pretreated with OM-MSCs and CUR-OM-MSCs before Hemin-neuron-CMs insult. (**A-H**) The levels of oxidative stress in OM-MSCs and CUR-OM-MSCs, (**A**) ROS, (**B**) ATP, (**C**) LPO, (**D**) MDA, (**E**) T-AOC, (**F**) T-SOD, (**G**) T-GSH, (**H**) GSH/GSSG ratio. (**I**) The cell viability of OM-MSCs and CUR-OM-MSCs. (**J**) The LDH level of OM-MSCs and CUR-OM-MSCs. Data are expressed as the mean ± SEM (n = 3), *P < 0.05, **P < 0.01, ***P < 0.001; ^#^P < 0.05, ^##^P < 0.01, and ^###^P < 0.001 represent CUR-OM-MSCs group vs. relative OM-MSCs group.

To further confirm the role of anti-oxidative effects in the survival of CUR-OM-MSCs under the microenvironment triggered by ICH, the oxidative stress inducer (H_2_O_2_) and the oxidative stress-antioxidative mediator (NAC) were used in hemin-neuron-CMs treated OM-MSCs. First, the treatment with the oxidative stress-inducing agent (H_2_O_2_) was referred to as positive control. Compared to the CUR-OM-MSCs group, the ATP, T-AOC, T-SOD, and T-GSH levels were decreased (Fig. 8B–G; *p* < 0.05), and the ROS, LPO and MDA levels (Fig. 8A, C and 8D; *p* < 0.05) within CUR-OM-MSCs were significantly upregulated following hemin-neuron-CMs treatment in the CUR-OM-MSCs + H_2_O_2_ group. No significance was found in the level of GSH/GSSG (Fig. 8H; *p* > 0.05). With the aggravation of oxidative stress, the cellular viability was decreased, and the LDH level of OM-MSCs was enhanced in the CUR-OM-MSCs + H_2_O_2_ group, relative to the CUR-OM-MSCs group (Fig. 8I and J; *p* < 0.01, *p* < 0.05, respectively). Second, we incubated OM-MSCs with NAC for 24 h prior to the treatment with hemin-neuron-CMs. As shown in the results, there was a marked increase in anti-oxidative capacities in OM-MSCs and CUR-OM-MSCs after NAC treatment. This was presented with elevated ATP, T-AOC, T-SOD, and T-GSH levels (Fig. 8B–G), and suppressed ROS, LPO and MDA levels (Fig. 8A, C and 8D) in the hemin-neuron-CMs + OM-MSCs/CUR-OM-MSCs + NAC group, compared to the relative hemin-neuron-CMs + OM-MSCs/CUR-OM-MSCs group. No significance was found in the level of GSH/GSSG (Fig. 8H; *p* > 0.05). Furthermore, with the alleviation of oxidative stress, the cellular viability was increased, and LDH level was significantly mitigated in the hemin-neuron-CMs + CUR-OM-MSCs + NAC group, relative to the hemin-neuron-CMs + CUR-OM-MSCs group (Fig. 8I and J; *p* < 0.001, *p* < 0.01, respectively). Moreover, we also compared the indexes of oxidative stress and survival between OM-MSCs and CUR-OM-MSCs groups. Under NAC treatment, the ROS, LPO and MDA levels in the CUR-OM-MSCs group were significantly lower than in the OM-MSCs group (Fig. 8A, C and 8D; *p* < 0.05). Accordingly, the indexes of cellular survival in the CUR-OM-MSCs group were also significantly ameliorated relative to the OM-MSCs group (Fig. 8I; *p* < 0.01).

Taken together, these observations implied that curcumin pretreatment augmented the survival of OM-MSCs in the microenvironment of ICH by upregulating anti-oxidative capacities of OM-MSCs.

## 4 Discussion

Currently, ICH remains difficult to completely cure subtype of cerebral stroke attributed to complicated pathophysiological mechanisms [26]. After ICH, the hematoma and its degradation substances initiated a persistent oxidative stress response that overwhelms the cellular antioxidant defense, eventually resulting in permanent neuronal death, BBB dysfunction, and aggravated secondary neurological injury [27]. Ferroptosis, characterized by an iron-dependent subtype of oxidative cell death, has been recently identified [28]. Consisted with the previous reports, the current results confirmed that ferroptotic neuronal death could also occur in the ICH model in vivo and vitro, including remarkedly alteration ferroptosis associated with protein expression, and lipid peroxidation indexes, as well as the typical dysfunction of mitochondrial morphology in the insult neurons. Meanwhile, it has been reported that the suppressor of ferroptosis, such as Ferrostatin-1, exerted neuroprotective effects on ICH-triggered neuronal death [7]. Hence, exploring novel methods to inhibit oxidative stress reactions triggered by ICH impairment is vital, thereby rescuing the ferroptotic neuronal death and neurological function deficit.

As of yet, the satisfactory and available treatment therapies for acute or long-term ICH patients remain extremely limited. MSCs transplantation provided potential promise in neuroprotection and neuronal restoration effects on ICH, attributed to its strong proliferation capacity, multipotent differentiation property, and unique paracrine effects [29]. Various types of tissues have been applied to isolate and harvest the MSCs, such as bone marrow, adipose, and placental. Whereas it is essential to note that although MSCs of various tissue-derived origins generally have similar properties, their multiple profiles, gene or protein expression patterns, and clinical application depend on their origin [30]. OM-MSCs isolated from olfactory mucosa tissues originated from the neural crest instead of traditional mesodermal sources [13]. Thus, OM-MSCs transplantation therapy might be more appropriate in treating neurology diseases, particularly cerebral vascular disorders. To verify the hypothesis, several studies have focused on the neuroprotection role of OM-MSCs in hemorrhagic or ischemic stroke. Our previous studies have found that OM-MSCs played key neuroprotective roles in cerebral stroke, including ICH, through restraining oxidative stress, and inflammation and enhancing angiogenesis, accordingly mitigating different forms of neuronal death, such as apoptosis and pyroptosis [[14], [15], [16],^31^]. In the present study, our data further confirmed the protected role of OM-MSCs in ferroptotic neuronal death initiated by the ICH model *in vitro* and in vivo. The results observed that OM-MSCs treatment could ameliorate ferroptosis caused by ICH injury in primary neurons and peri-hematoma brain tissues. Consistent with the previous reports [8], our data also identified that the MSCs-based method is an optimized therapy for neuronal death and recovery post-ICH.

At present, there are still some challenges for MSCs transplantation in ICH clinical practice. The interaction relationship between the complicated pathological microenvironment initiated by ICH and transplanted various MSCs, including OM-MSCs, should be highly considered and further investigated [32]. The microenvironment derived from the insulted neuronal exerted a critical effect on the survival, proliferation, differentiation, and senescence of engrafted MSCs [33]. Our previous study has demonstrated that the extracellular vesicles from the injured neuronal cell have significantly inhibited the survival of engrafted MSCs by promoting apoptosis and the levels of oxidative stress under cerebral ischemic/reperfusion conditions [34]. Therefore, it is urgent to explore the available method to enhance the survival of engrafted OM-MSCs upon harsh microenvironment initiated by ICH, thereby promoting the neuroprotective effect of OM-MSCs on the hemorrhage stroke.

Curcumin is extracted from curcuma longa, which played an important role in regulating oxidative stress, inflammation, and immunity response [35]. Multiple *in vitro* and in vivo experiments have been carried out to investigate the role and mechanism of curcumin in intracerebral hemorrhage and revealed that curcumin participates in the recovery of intracerebral hemorrhage injury by inhibiting the oxidation, apoptosis, and inflammation, protecting the BBB [18,19,36,37]. Some previous studies have suggested curcumin ameliorated ICH-induced brain tissue impairment and brain edema [17,38]. Whereas the clinical application of curcumin remains suffers from some potential challenges, including poor oral absorption and water solubility, and unable cross the blood-brain barrier [39]. Therefore, the current study hypothesized that curcumin co-cultured with OM-MSCs transplantation therapy might be provided with a novel method for ICH treatment. On the one hand, curcumin combined with OM-MSCs could compensate for the shortcomings of curcumin, thereby further improving the therapeutic effect of OM-MSCs. Moreover, the survival of engrafted OM-MSCs might be enhanced by the harsh microenvironment induced by ICH via the anti-oxidative effect on curcumin preconditioning.

As we expected, the present results observed that curcumin combined with OM-MSCs transplantation therapy could more obviously protect against the excessive oxidative stress and ferroptosis neuronal death caused by ICH injury, thereby mitigating the brain tissues insult and recovering the neurological deficit compared to the OM-MSCs single therapy. Considering the above data, we further evaluated whether the improvement of neuroprotective on CUR-OM-MSCs post-IHC is related to the enhanced survival of transplanted OM-MSCs. The present results observed a significantly enhanced cell viability and reduced LDH leakage of CUR-OM-MSCs co-cultured with a Hemin-treated conditioned medium. Moreover, the levels of oxidative stress were also remarkedly suppressed in the CUR-OM-MSCs co-cultured with Hemin treated conditioned medium. Consisted with a recent study, Lin et al. found that curcumin pretreatment could also improve the survival of Human Umbilical Cord-Derived MSCs and rescue the motor outcomes during spinal cord injury by the ERK1/2 signalling pathway [21]. These results indicated that pretreatment with curcumin could augment the neuroprotective effects on OM-MSCs after ICH-induced insult by abolishing ferroptosis and oxidative stress in neuronal death via elevating the survival of engrafted OM-MSCs upon the harsh microenvironment initiated by ICH.

The present study bears some clinical relevance, but there are limitations. First, although basic research has yielded positive data, it is essential to note that there are significant differences in CUR-OM-MSCs transplantation between animals and humans, such as dose, frequency of cell transplantation, route, and time point of treatment. Second, considering the previous studies showed that the DMSO had no effect on the characteristics of MSCs [40], we did not adopt the DMSO as a vehicle control in our study. Third, the issue regarding the treatment improvement effect by human stem cells or by the inflammation induced due to xenotransplantation remains unsolved. In the further study, we will compare the therapeutic efficacy, mechanisms, and immune response between the human MSCs and mouse MSCs. Moreover, considering the characteristic feature of curcumin, such as short half-life, nearly insolubility in water and lower bioavailability, the effect of CUR-OM-MSCs should be further illustrated to identify the possible molecular mechanism and the delivery efficiency, stability, and safety of their synergistic function role in improving the therapeutic effect of cerebral hemorrhage. Therefore, further research needs to be conducted.

## 5 Conclusions

Curcumin, a crucial ingredient of turmeric, has been identified to play a significant role in the therapy of cerebral hemorrhage via various mechanisms such as anti-oxidation, anti-neuronal death and brain tissue insult induced by ICH. This current research exhibited that curcumin co-cultured with OM-MSCs transplanted therapy was more effective in preventing ferroptotic neuronal death and brain tissue impairment caused by ICH *in vitro* and in vivo. This novel combination strategy might be provided with a promising cell-based candidate treatment for clinical application in the ICH challenge.

## Funding statement

These whole experiments were supported by the National Nature Science Foundation of China (Grant no. 82101544, 61972460, 62172440, 61802443), the National Natural Science Foundation of Hunan Province (Grant no. 2019JJ20037, 2020JJ4883, 2020JJ4923, 2021JJ40368, 2023JJ30331), National Key Research and Development Program of Hunan Province (Grant no. 2021sk2023) and Key Project of Hunan Provincial Maternal and Child Health Care Hospital (Grant no. 2021RX01).

## Ethics statement

All animal procedures were performed in accordance with the Guide for the Care and Use of Experimental Animals, and were approved by the Animal Care and Use Committee of Hunan Provincial Maternal and Child Health Care Hospital (Approved No.2021-S010). Human OM-MSCs were obtained from healthy donors (four males, aged 20–40 years old) at the Second Affiliated Hospital of Hunan Normal University (Changsha, Hunan, China). The ethics committee of the Hunan Normal University has approved this procedure protocol (Approved No.2009163009). Consent for publication is Not applicable.

## Author contribution statement

Yan Huang: Conceived and designed the experiments; Analyzed and interpreted the data; Wrote the paper.

Jianyang Liu, Jialin He: Analyzed and interpreted the data; Contributed reagents, materials, analysis tools or data.

Fengbo Tan, Ming Lu, Fulai Yuan, Xuelin Zhu: Performed the experiments.

Lingyu Kong: Conceived and designed the experiments; Contributed reagents, materials, analysis tools or data; Wrote the paper.

## Data availability statement

Data included in article/supp. Material/referenced in article.

## Declaration of competing interest

The authors declare that they have no known competing financial interests or personal relationships that could have appeared to influence the work reported in this paper.

## Figures and Tables

**figs1:**
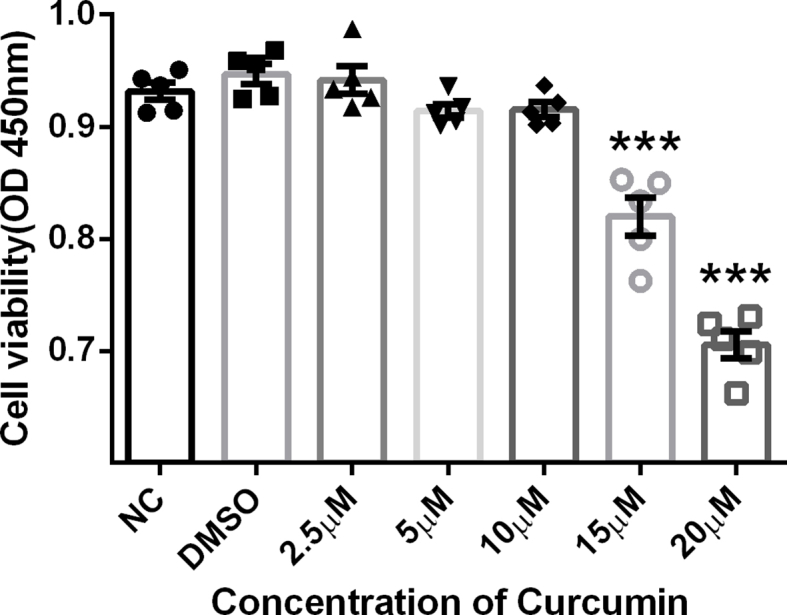


**figs2:**
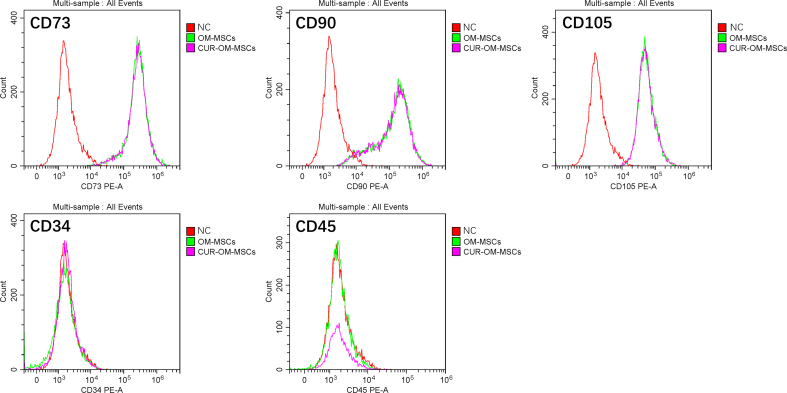


**figs3:**
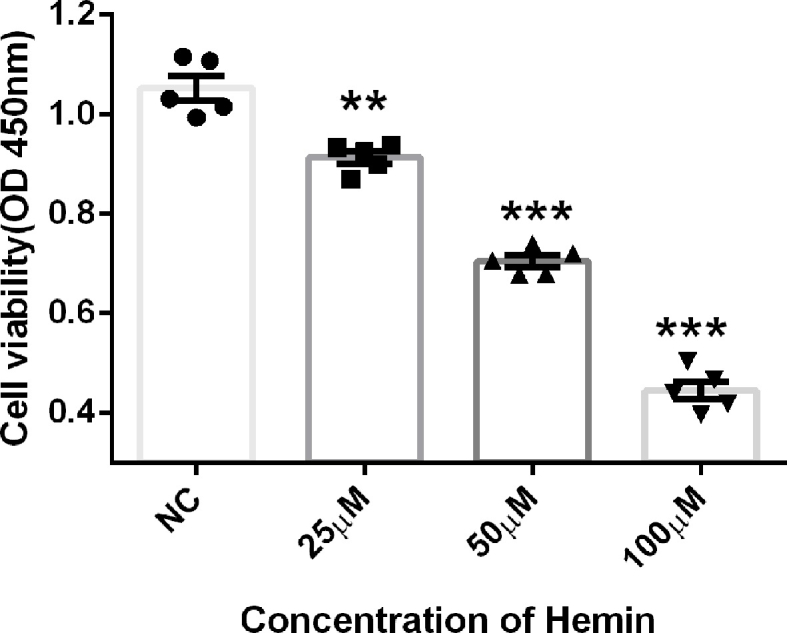


**figs4:**
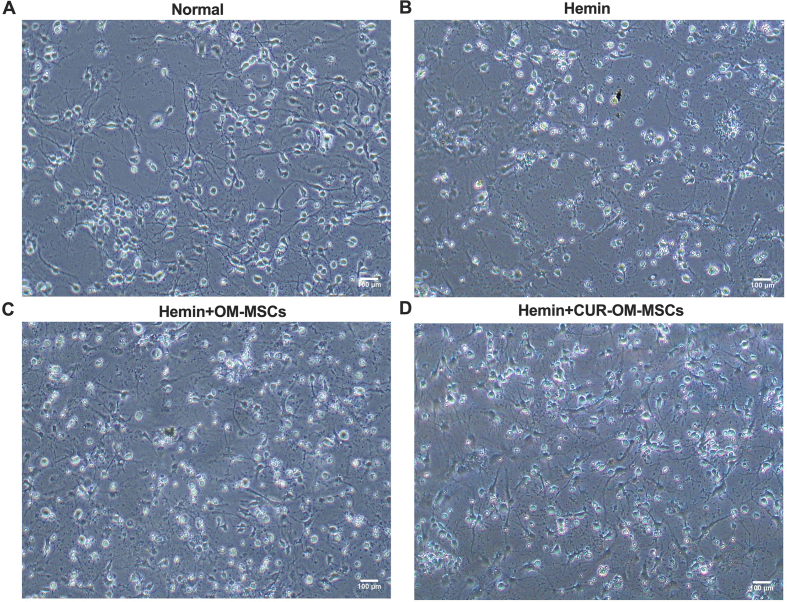


**figs5:**