# Supplementary figures and images for: Curcumin preconditioning enhances the neuroprotective effects of olfactory mucosa-derived mesenchymal stem cells on experimental intracerebral hemorrhage
Source: Heliyon. 2023 Jul 3;9(7):e17874. doi: 10.1016/j.heliyon.2023.e17874 (PMC10359873; doi:10.1016/j.heliyon.2023.e17874)

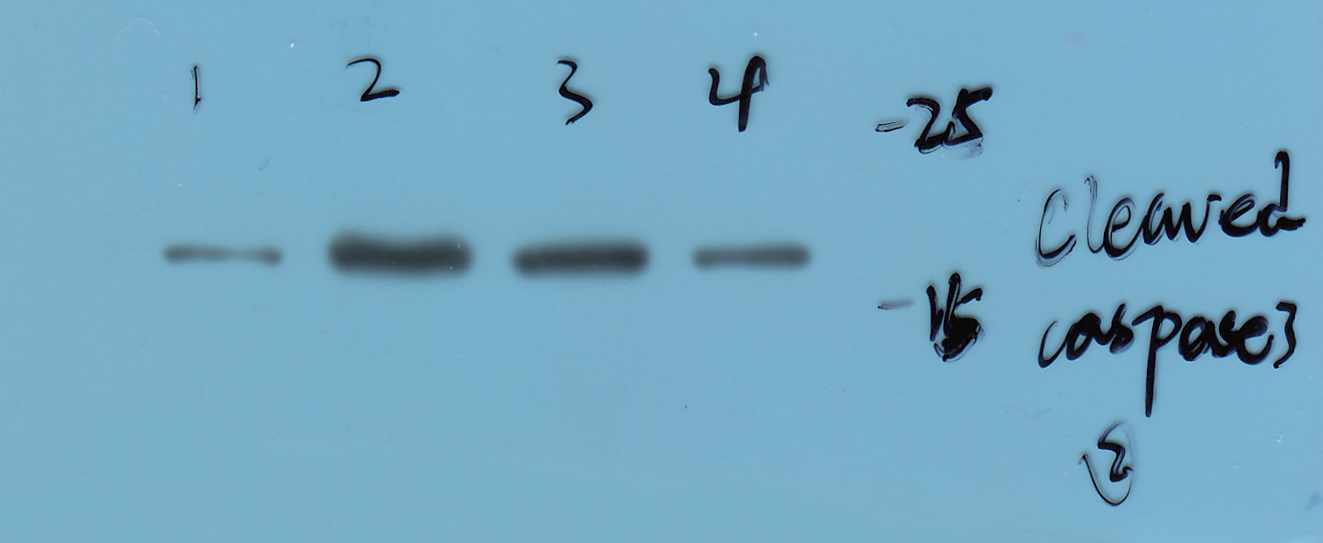

Supplement: Multimedia component 1 [file mmc1.zip › Figure 2G/cleaved-caspase3.tif]

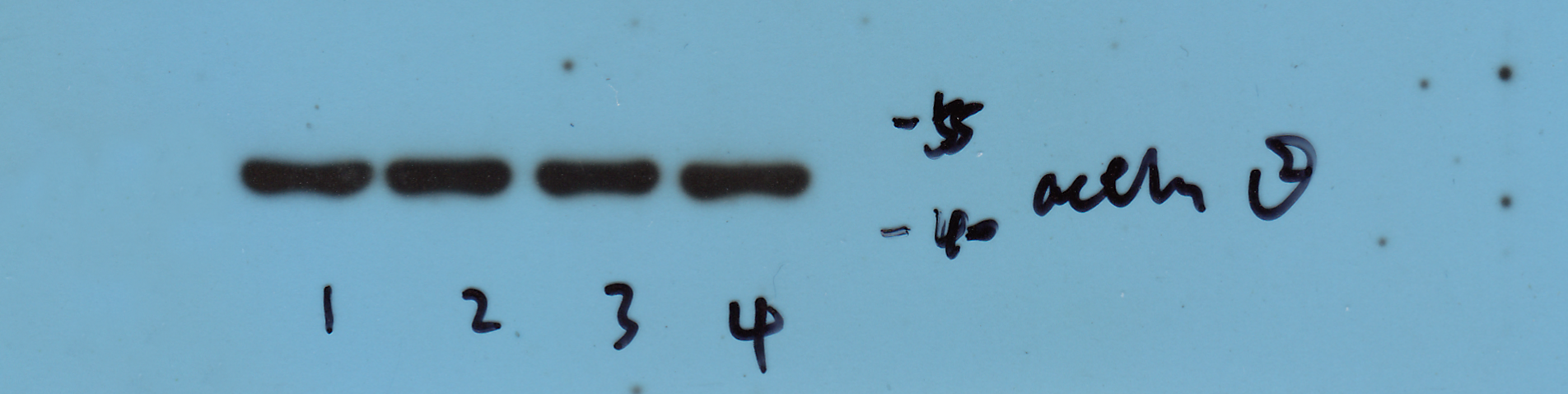

Supplement: Multimedia component 1 [file mmc1.zip › Figure 2G/β-actin.tif]

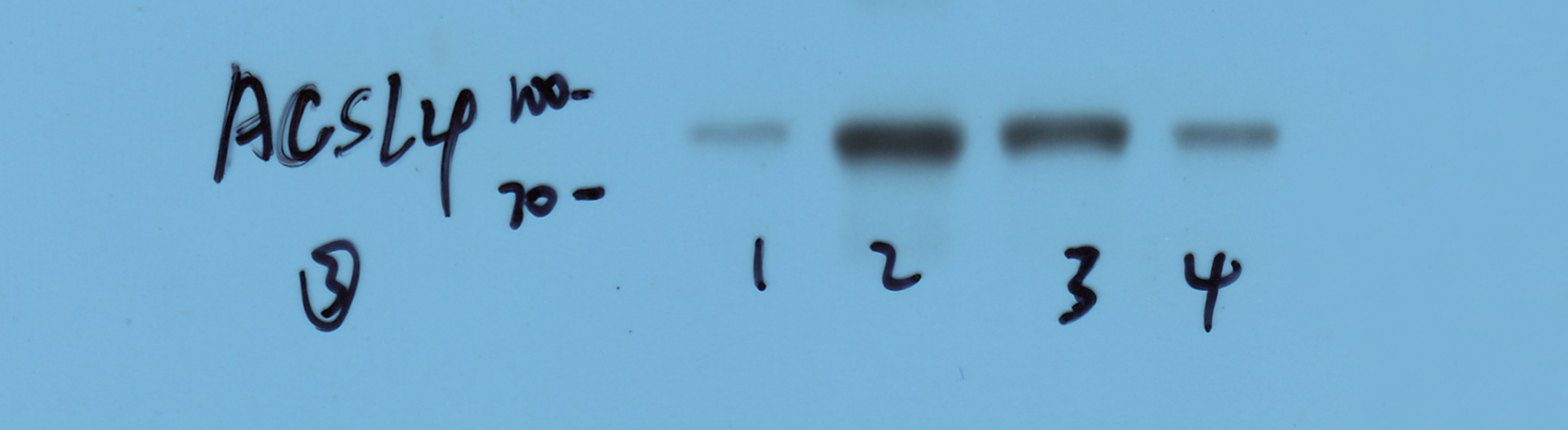

Supplement: Multimedia component 1 [file mmc1.zip › Figure 3K/ACSL4.tif]

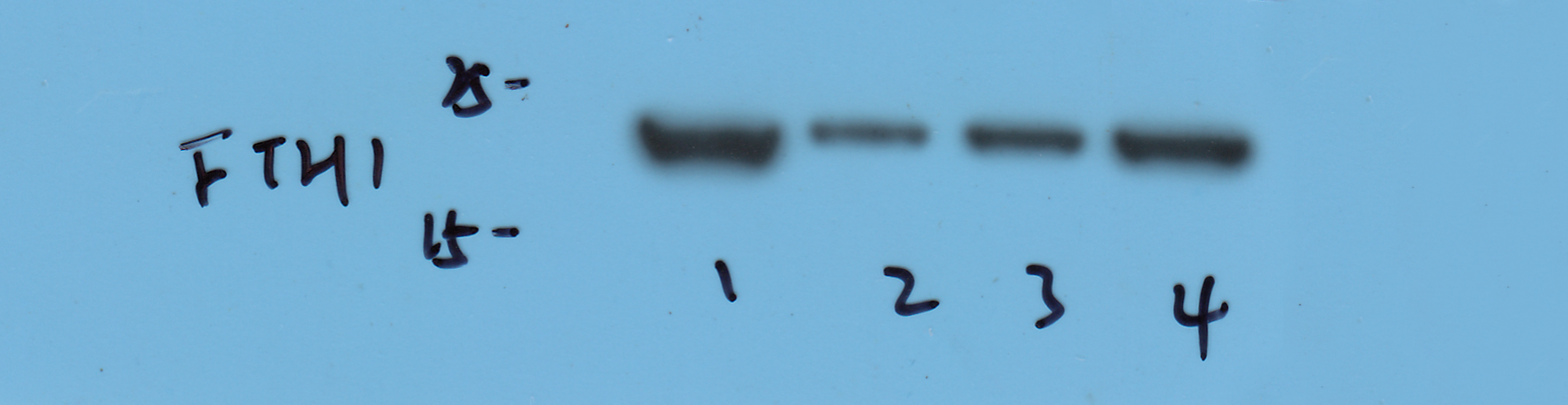

Supplement: Multimedia component 1 [file mmc1.zip › Figure 3K/FTH1.tif]

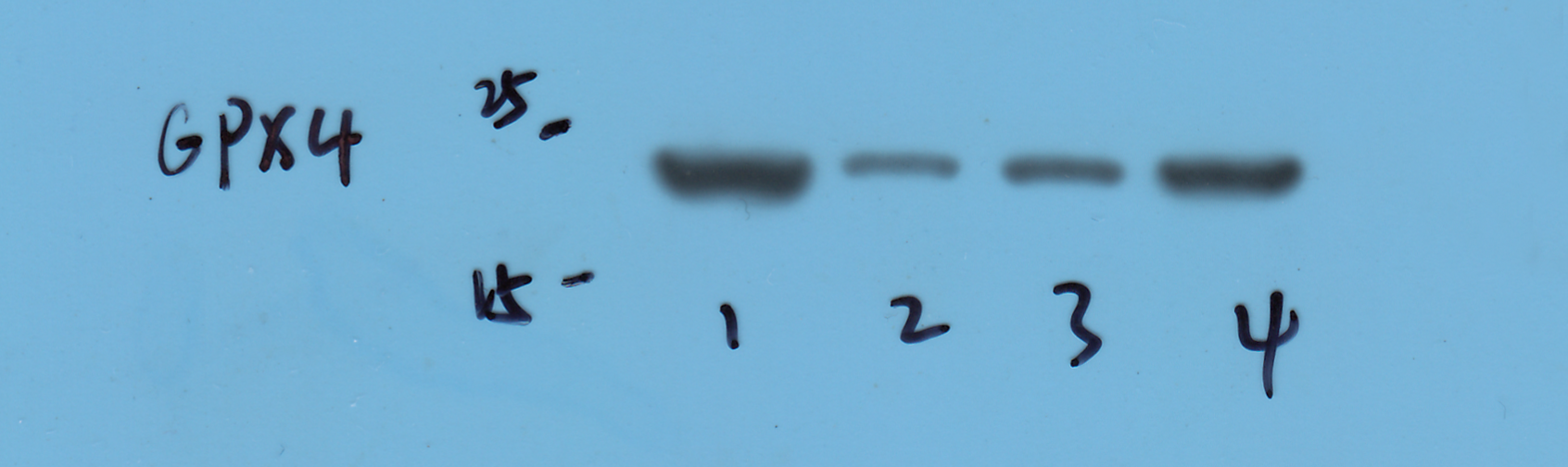

Supplement: Multimedia component 1 [file mmc1.zip › Figure 3K/GPX4.tif]

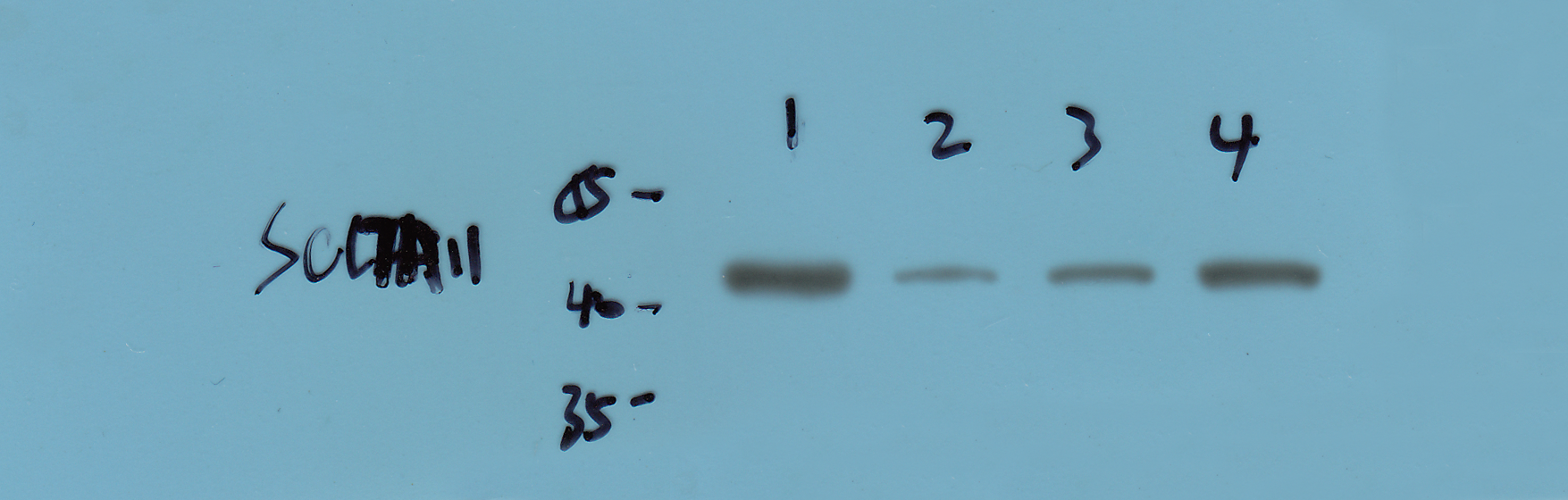

Supplement: Multimedia component 1 [file mmc1.zip › Figure 3K/SCL7A11.tif]

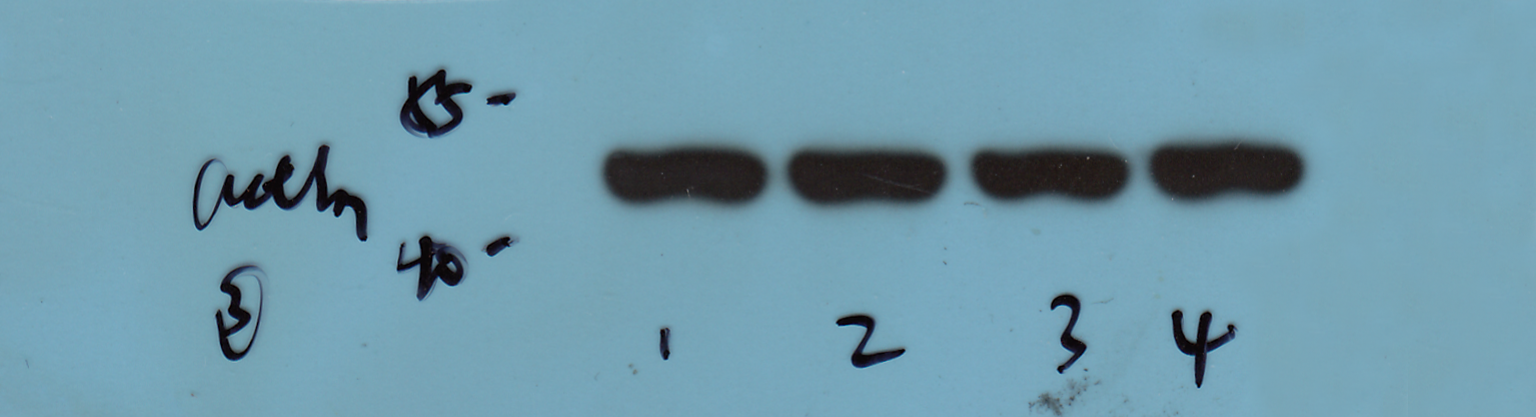

Supplement: Multimedia component 1 [file mmc1.zip › Figure 3K/β-actin.tif]

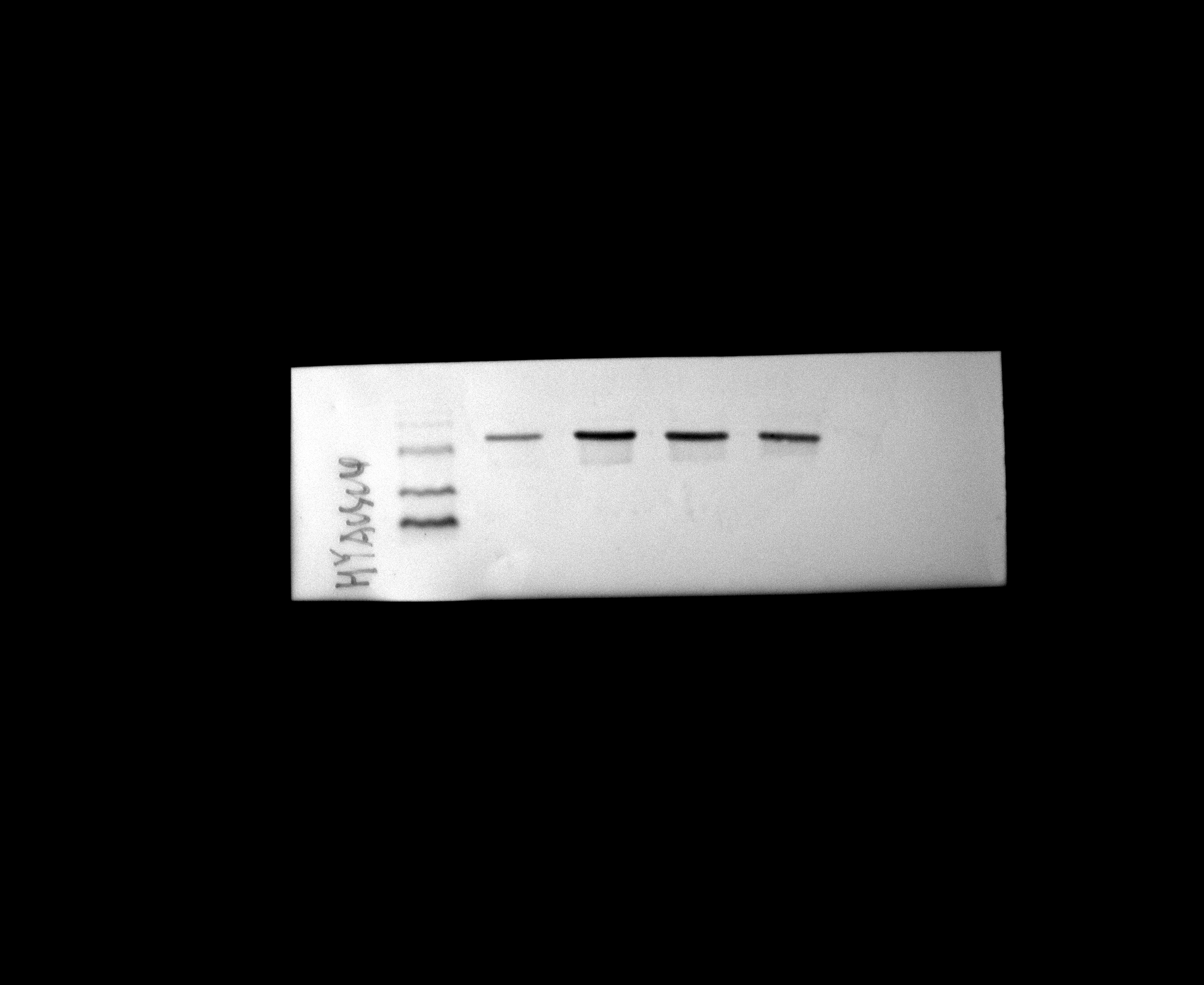

Supplement: Multimedia component 1 [file mmc1.zip › Figure 5C/ACSL4.tiff]

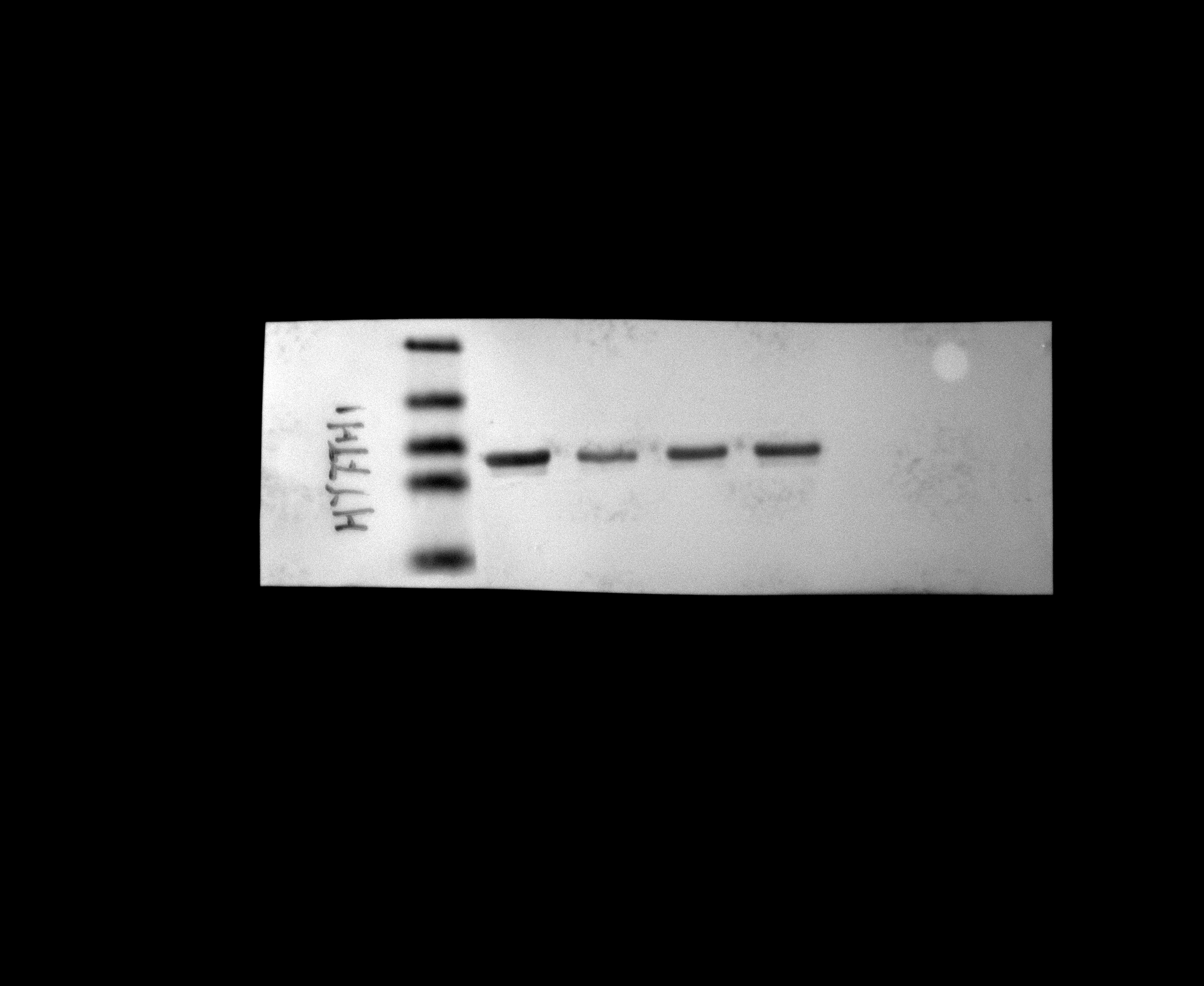

Supplement: Multimedia component 1 [file mmc1.zip › Figure 5C/FTH1.tiff]

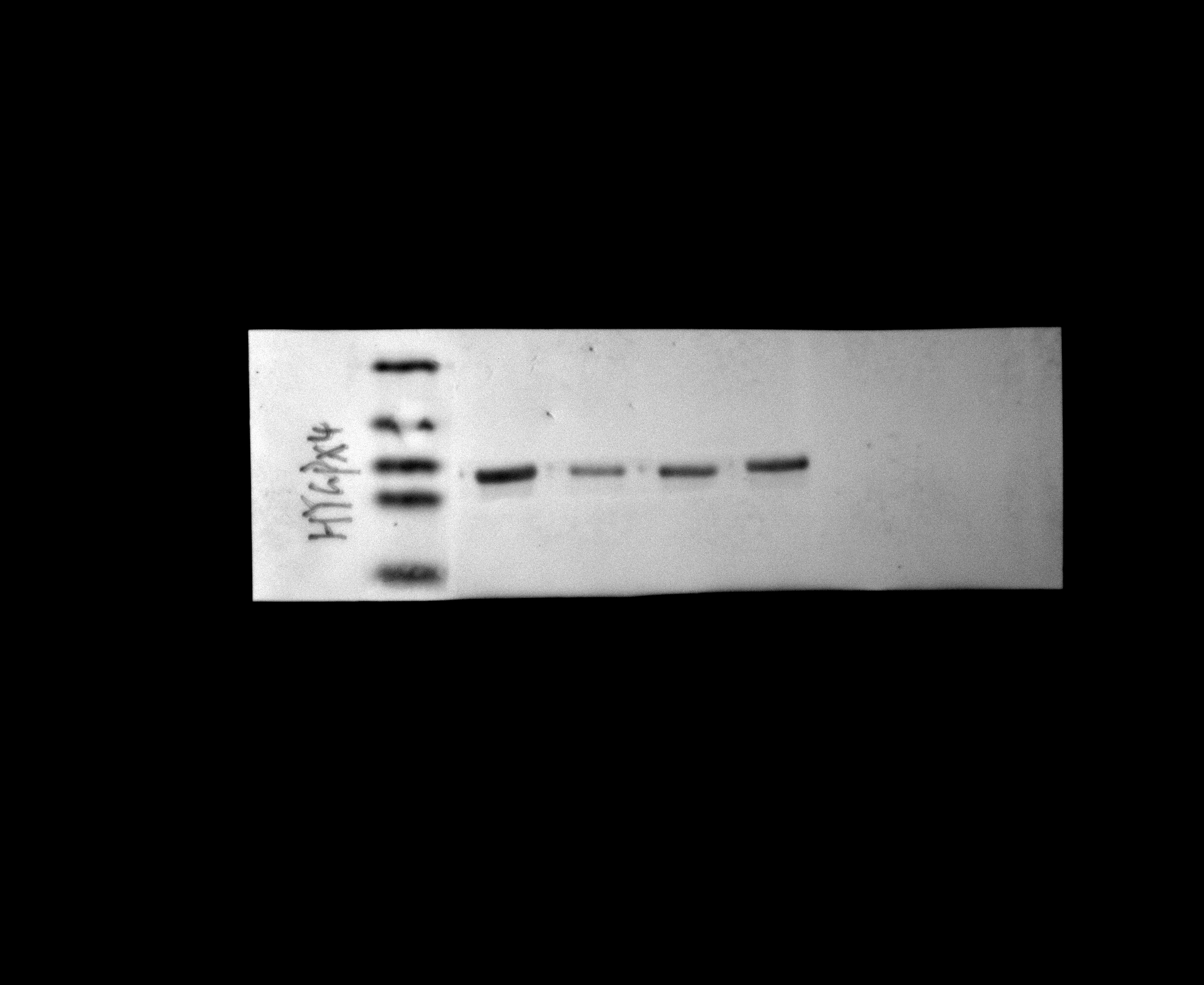

Supplement: Multimedia component 1 [file mmc1.zip › Figure 5C/GPX4.tiff]

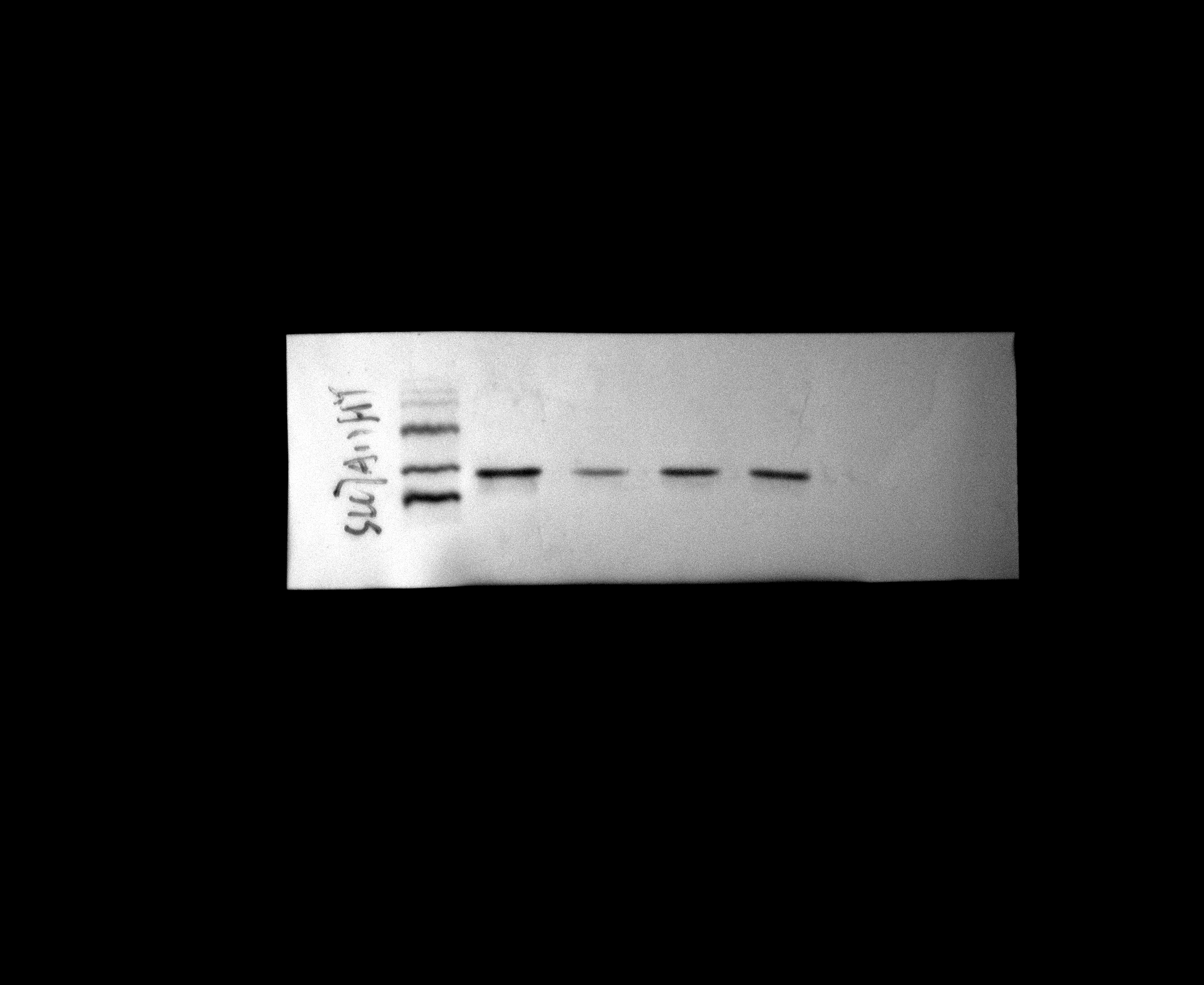

Supplement: Multimedia component 1 [file mmc1.zip › Figure 5C/SLC7A11.tiff]

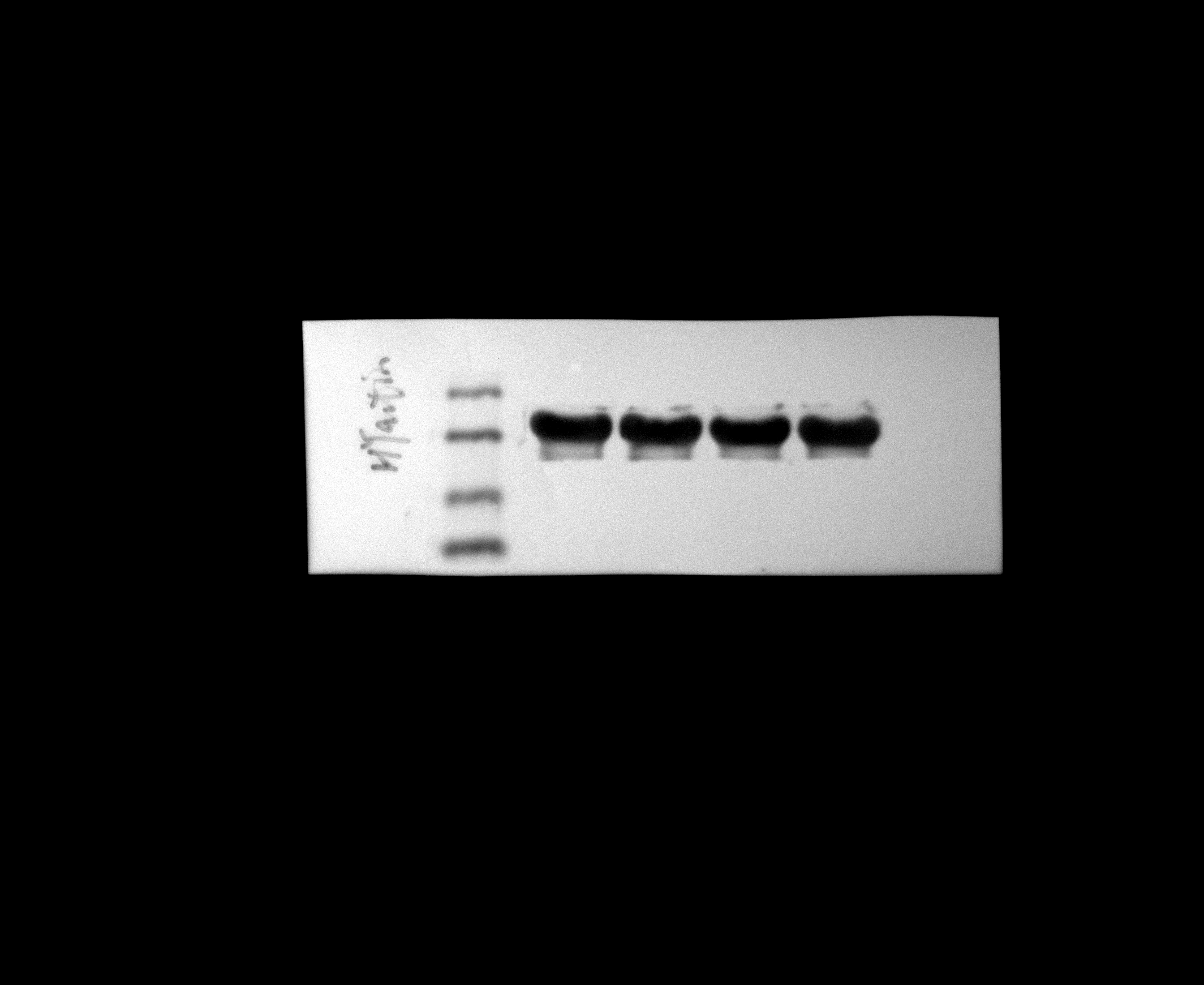

Supplement: Multimedia component 1 [file mmc1.zip › Figure 5C/β-actin.tiff]

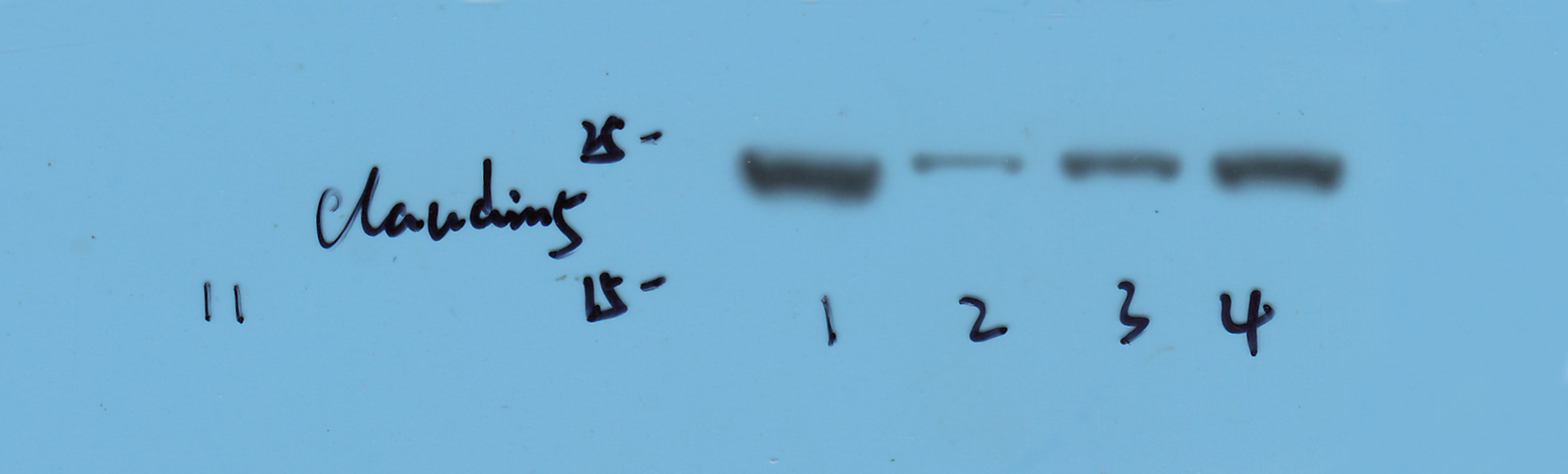

Supplement: Multimedia component 1 [file mmc1.zip › Figure 6B/claudin-5.tif]

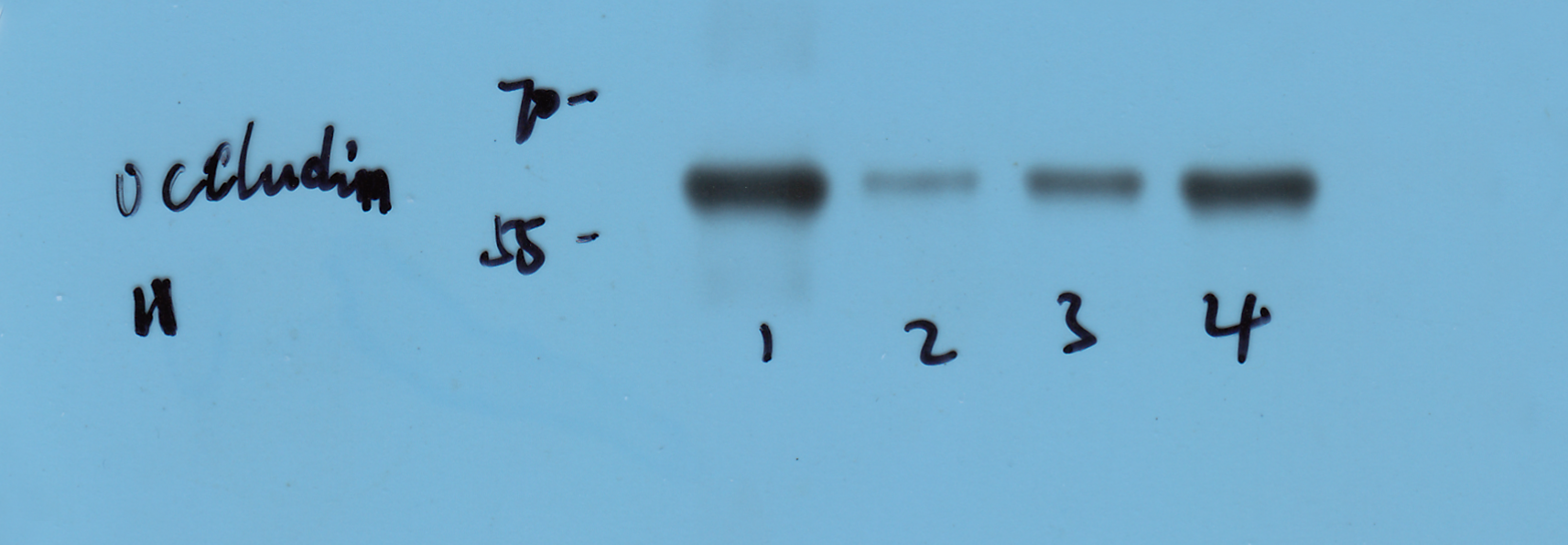

Supplement: Multimedia component 1 [file mmc1.zip › Figure 6B/Occludin.tif]

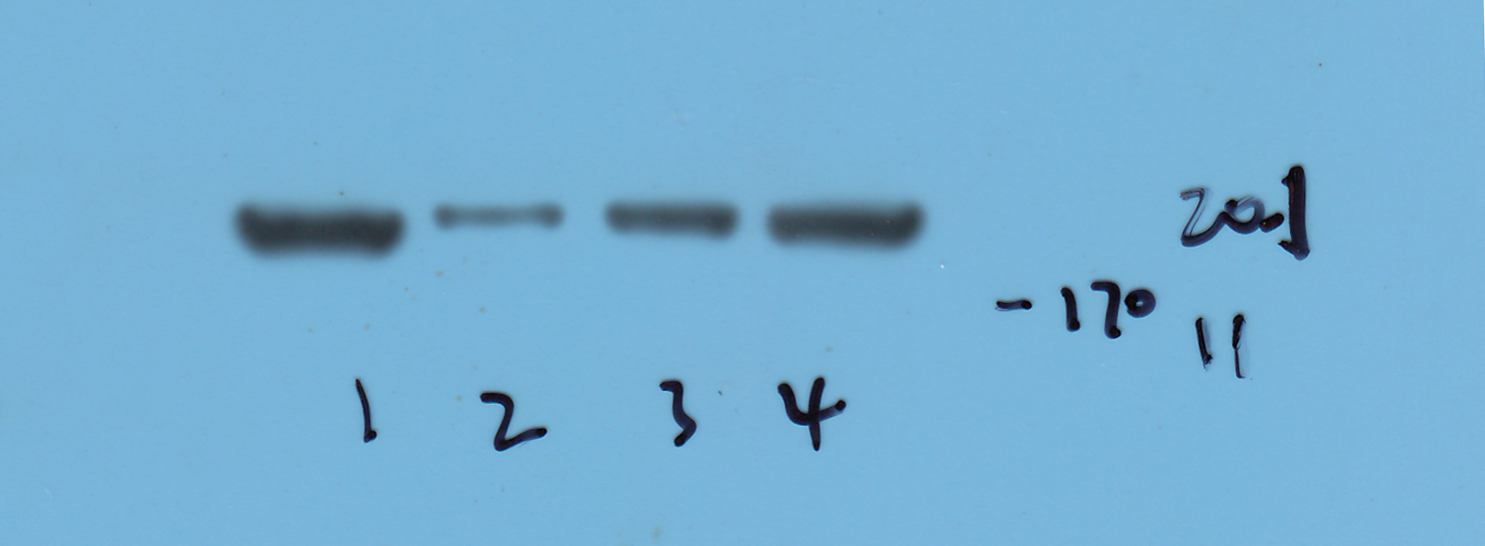

Supplement: Multimedia component 1 [file mmc1.zip › Figure 6B/zo-1.tif]

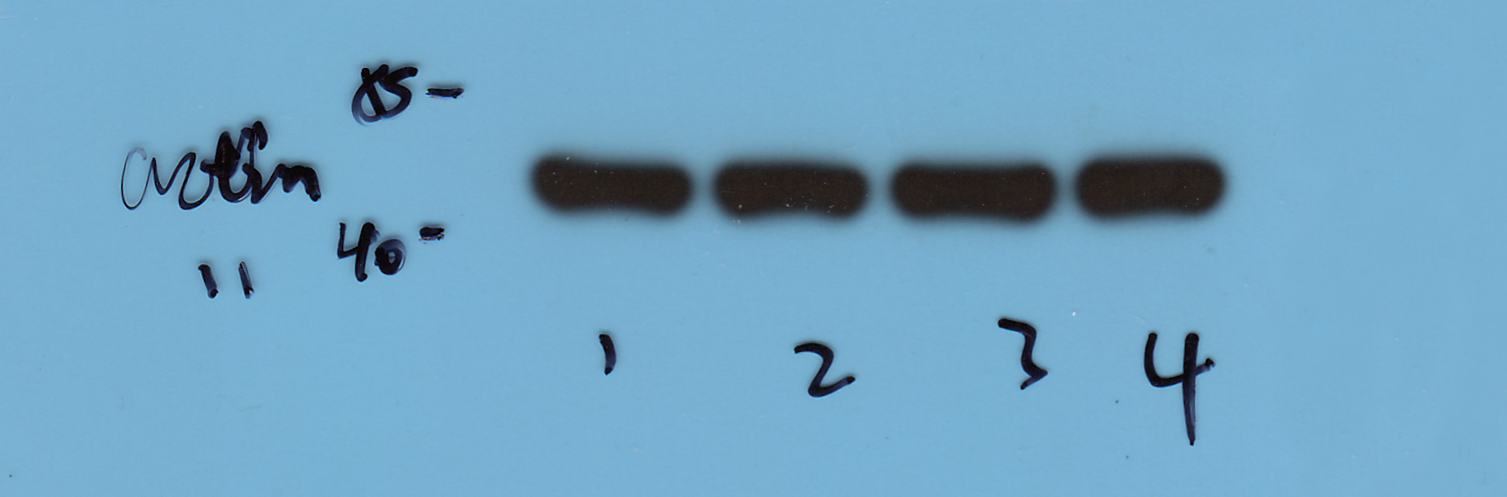

Supplement: Multimedia component 1 [file mmc1.zip › Figure 6B/β-actin.tif]
